# Elucidating the Covalent Binding of Ottelione A and Withaferin A at the Colchicine Site of β‑Tubulin

**DOI:** 10.1021/acsomega.6c05137

**Published:** 2026-08-31

**Authors:** Mario Carreón-Escalante, Efrén Mar-Antonio, Samuel Sánchez-Maza, Hugo A. Salgado-Flores, Rodrigo Aguayo-Ortiz

**Affiliations:** Departamento de Farmacia, Facultad de Química, Universidad Nacional Autónoma de México, Mexico City 04510, Mexico

## Abstract

Ottelione A (OTT) and withaferin A (WIT) are natural products known to induce microtubule depolymerization by covalently modifying β-tubulin within the colchicine binding site (COLbs). Experimental evidence suggests that both compounds primarily react with βCys239 through Michael-type additions. In this work, orbital-weighted dual descriptor analysis, relative stability of methyl thiolate adducts, ensemble covalent docking, and molecular dynamics simulations were employed to investigate their reactivity and covalent binding modes. While initial reactivity analysis identified multiple electrophilic centers in both ligands, OTT shows a thermodynamic preference for 1,6-addition products due to hyperconjugative stabilization, whereas WIT favors 1,4-addition products over endergonic 1,2-addition. Docking and molecular dynamics simulations are consistent with these observations, as OTT preferentially forms 1,6-Michael adducts within the COLbs, while WIT forms 1,4-Michael adducts and can adopt an alternative conformation oriented toward the αβ-tubulin interface. Notably, this pose promotes displacement of the β-T7 loop and increased solvent exposure of the hydrophobic COLbs pocket, suggesting a potential structural basis for tubulin destabilization. Overall, this study provides molecular insight into the covalent interaction of OTT and WIT with β-tubulin and suggests structural features underlying their microtubule-destabilizing activity.

## Graphical abstract

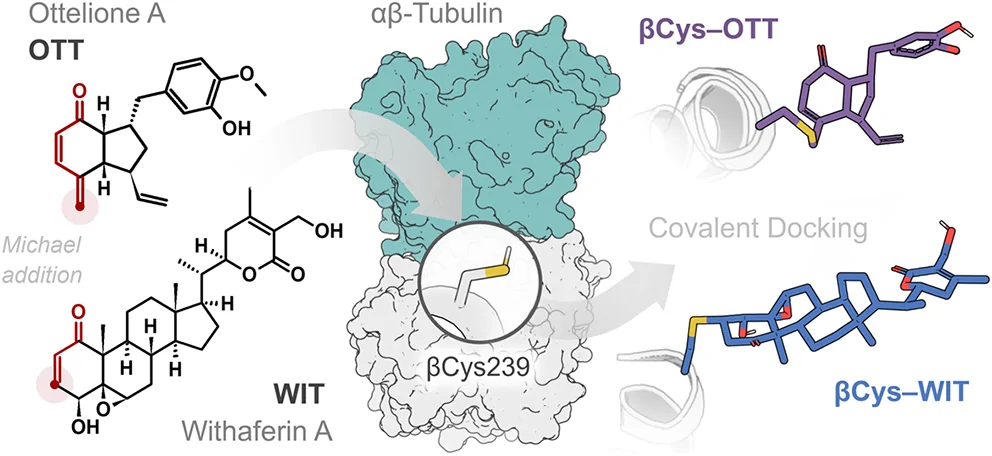

## Introduction

Natural products (NPs) continue to represent a fundamental source of lead compounds in the development of therapeutics for both human and veterinary medicine, underscoring their enduring relevance in contemporary drug discovery.^1–3^ Over the past century, a wide range of NPs have been identified as microtubule-targeting agents (MTAs), many of which have been shown to disrupt mitotic progression in cancer cell lines.^4^ These include microtubule-destabilizing agents such as colchicine (COL), combretastatin A4 (CA-4), vinblastine, and maytansine, as well as microtubule-stabilizing agents, including paclitaxel, laulimalide, and epothilone A.^5^ Furthermore, the binding sites of these and other NPs have been mapped on the αβ-tubulin heterodimer subunits of microtubules.^6^ Among compounds with characterized binding sites, only three NPs have been shown to bind covalently to tubulin: pironetin, taccalonolide AJ, and zampanolide.^7–9^ Pironetin exerts its microtubule-destabilizing effect by forming a covalent bond with Cys316 of α-tubulin, thereby disrupting the longitudinal contacts between tubulin heterodimers.^7^ Conversely, taccalonolide AJ and zampanolide stabilize microtubules through covalent interactions with Asp224 and His227 of β-tubulin, respectively, inducing a conformational arrangement of the M-loop that enhances lateral contacts between adjacent tubulin subunits.^8,9^ Notwithstanding these advances, the exact binding sites and mechanisms of action of numerous covalently interacting NPs have yet to be fully elucidated.^10,11^ Among the few exceptions are ottelione A (OTT) and withaferin A (WIT) (Figure 1A), whose covalent binding to β-tubulin and microtubule-depolymerizing activity have been well characterized. Both molecules interact with COL binding site (COLbs) on β-tubulin, located near the intradimer interface with the α-tubulin monomer, and are likely to occupy one or more of the three zones (Z1 to Z3) described for this site (Figure 1A).^12,13^

**Figure 1:**
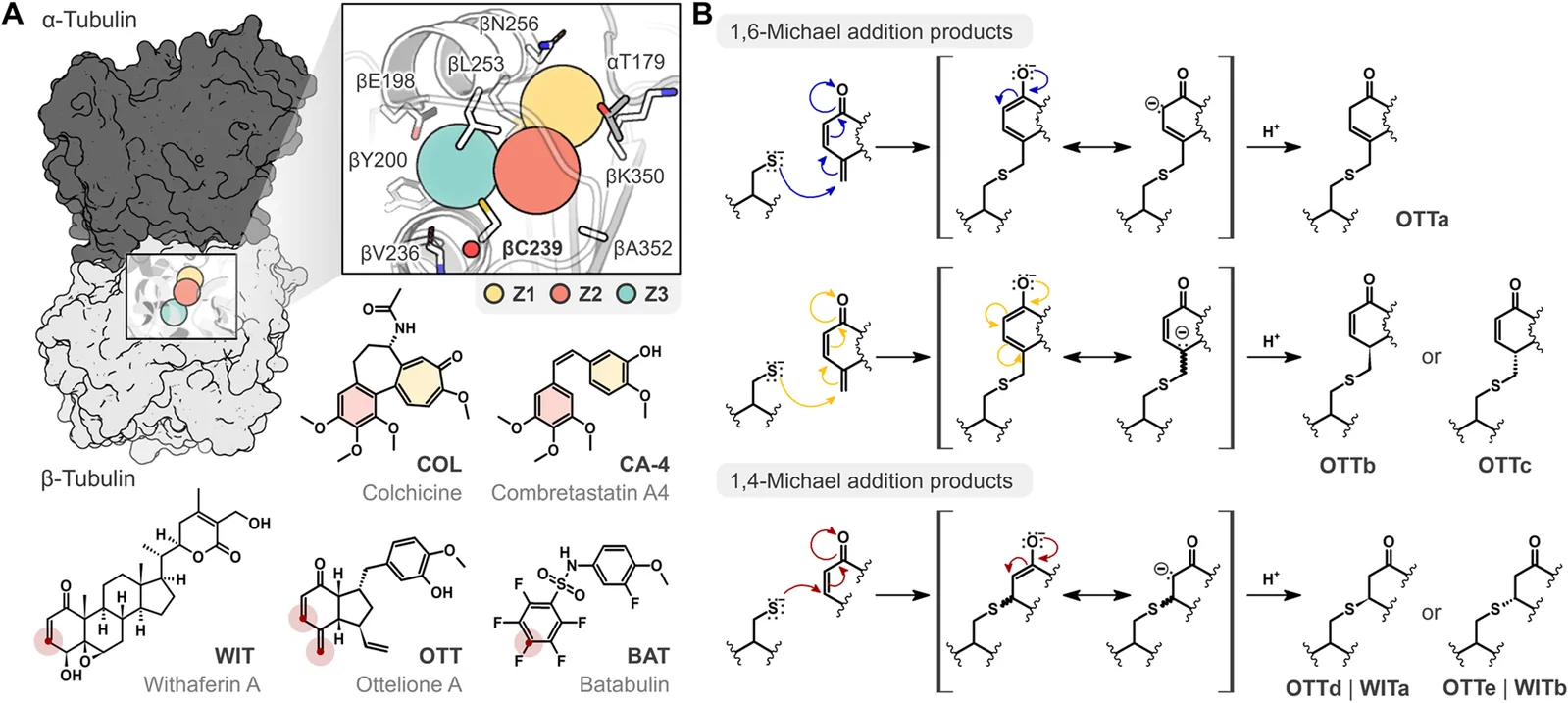
(A) Depiction of the αβ-tubulin heterodimer (PDB ID: 4O2B^25^) highlighting the colchicine-binding site (COLbs), the three subregions that constitute this pocket (Z1 to Z3), and the reactive cysteine residue βCys239 (βC239). Figure modified from Colorado-Pablo et al.^13^ At the bottom, the chemical structures of ottelione A (OTT) and withaferin A (WIT), along with the synthetic compound batabulin (BAT), are shown. For reference, colchicine (COL) and combretastatin A-4 (CA-4) chemical structures are also presented with color coding based on the COLbs zones. For WIT, OTT, and BAT, the region most susceptible to nucleophilic attack by βCys239 is highlighted in red. (B) Schematic representation of the proposed reaction pathways leading to product formation through 1,6-Michael addition (top) and 1,4-Michael addition (bottom). The depicted products correspond to the keto forms obtained either by protonation of the Cα carbanion or by protonation of the enolate oxygen followed by keto-enol tautomerization.^26,27^ Each product derived from OTT and WIT was assigned a unique code.

OTT is a 4-methylene-2-cyclohexanone derivative whose chemical structure was first patented in 1996 under the codename RPR-112378 by Rhône-Poulenc Rorer S.A.^14^ A few years later, Ayyad et al.^15^ reported the isolation and structural elucidation of OTT from a diastereomeric mixture with ottelione B, both obtained from the nonpolar extract of the freshwater plant *Ottelia alismoides*, collected from the Nile River Delta in Egypt. The study also revealed that OTT exhibited *in vitro* antiproliferative activity against a panel of approximately 60 human cancer cell lines. This cytotoxicity observed for OTT has been attributed to its ability to inhibit tubulin polymerization.^16^ Further studies demonstrated that this compound competitively inhibits COL binding to tubulin, but not vinblastine binding, and suggested that its activity may involve a thiol-addition reaction with cysteine residues located within the COLbs.^17^ Moreover, antiproliferative activity studies with OTT analogs have suggested that the covalent interaction may occur through a 1,6-Michael addition at the exocyclic methylidene group of the molecule, as its removal of this functional group resulted in the loss of activity in the corresponding analog (Figure 1B).^18^ Based on these findings, cysteine at position 239 of β-tubulin (βCys239) has been proposed as the likely site of covalent and irreversible interaction with OTT.

WIT is a withanolide featuring an ergostane core fused with a δ-lactone ring, extracted from the plant *Withania somnifera*.^19^ Similar to OTT, WIT has demonstrated potential antitumor activity against various human cancer cell lines.^20,21^ Early studies suggested that both the α,β-unsaturation in ring A and the epoxide group contributed to its cytotoxic activity, presumably through covalent binding to microtubules.^22^ However, more recent investigations have shown that its antiproliferative effects are exclusively attributed to the reactivity of the α,β-unsaturated bond, likely through a 1,4-Michael addition with cysteine residues (Figure 1B).^23^ This covalent interaction of WIT with the αβ-tubulin heterodimer has also been linked to tubulin degradation via the intracellular ubiquitin-proteasome system.^23,24^ Although WIT-induced tubulin degradation was initially attributed to covalent binding to the cysteine residue at position 303 of β-tubulin (βCys303), subsequent studies with β-tubulin isotypes containing βCys303 but harboring a serine at position 239 (βIII and βVI isotypes) challenged this hypothesis.^23^ The results suggested that βCys239 at the COLbs, rather than βCys303, is the critical residue for covalent interaction and subsequent tubulin degradation. This conclusion was further confirmed using β-tubulin mutants C239S and C303S, which revealed that tubulin degradation occurred only in cell lines expressing wild-type β-tubulin or the C303S mutant, but not in those carrying the C239S mutation. Therefore, as in the case of OTT, the evidence supports that WIT exerts its activity through covalent binding to βCys239.

Similar to OTT and WIT, a few synthetic ligands with antiproliferative activity against human cancer cell lines have been reported to react with βCys239, occupying the COLbs on β-tubulin.^28^ Some of these compounds have been shown to induce conformational destabilization of the tubulin heterodimer, thereby promoting its degradation via the ubiquitin-proteasome system, as observed with WIT, thus coining the name of microtubule degradation agents (MDgAs).^29^ Representative examples include T0070907, T007-1, and T138067 or batabulin (BAT; Figure 1A), from which only the latter has been structurally characterized in complex with tubulin via X-ray crystallography.^30^ BAT irreversibly binds with βCys239 residue of β-tubulin through a nucleophilic aromatic substitution at position 4 of its pentafluorophenyl substituent. MDgAs such as BAT have emerged as a promising therapeutic strategy, not only because of their novel mode of action but also due to their potential to overcome multidrug resistance (MDR) as a consequence of their proteasome-mediated degradation pathway.^31^ Since tubulin selective degradation by small molecules was not identified as a mechanism of action until 2009 by Chung and colleagues,^32^ it is plausible that other βCys239-binding compounds may act through a similar pathway. Given the therapeutic relevance of MDgAs, elucidating the binding mode of previously reported βCys239-targeting ligands such as OTT and WIT with β-tubulin remains a critical objective.

In this study, we performed an ensemble covalent docking of both OTT and WIT targeting βCys239 across 138 tubulin structures deposited in the Protein Data Bank (PDB). To guide the docking studies, the reactivity of the ligands was assessed by calculating the orbital-weighted dual descriptor to identify their electrophilic atoms, followed by thermodynamic analysis of methyl thiolate adducts to confirm their most probable reactive site. Based on the top ensemble covalent docking results and consistency with experimental data, molecular dynamics simulations of the tubulin-ligand complexes were performed to characterize the potential orientation of ligands covalently bound to βCys239 within the COLbs. Furthermore, a putative mechanism by which WIT may induce tubulin degradation is proposed. Overall, these findings lay the groundwork for understanding the covalent mechanism of action of both OTT and WIT and for the development of new agents targeting βCys239.

## Theoretical Methods

### Quantum Methods

The 3D structures of OTT and WIT were generated from their isomeric SMILES using OpenBabel v3.1.1.^33^ Geometry optimizations and electronic structure calculations for both molecules were performed with Gaussian v16,^34^ using Density Functional Theory (DFT) and the SMD implicit solvation model for water.^35^ To address the possible differences in chemical accuracy, five different functionals were employed with the def2-TZVP^36,37^ basis set, namely: PBE0,^38^ B3LYP,^39–41^ CAM-B3LYP,^42^ M06-2X,^43^ and ωB97X-D.^44^ The Grimme’s D3 dispersion correction with Becke-Johnson damping^45^ (GD3-BJ) was used with PBE0 and B3LYP functionals, as neither include long-range electron correlation effects. Vibrational analysis was performed to verify that the optimized structures corresponded to true minima of the potential energy surface (PES). The orbital-weighted dual descriptor^46^ (Δ*f*_w_) was computed with Multiwfn v3.8,^47,48^ using a Gaussian function width (Δ) of 0.1 au to define the orbital weighting factors. The condensed-to-atom values of Δ*f*_w_ were determined from the Hirshfeld charges of *N* and *N ±* 1 electron states within the finite-differences approach.^49^

Methyl thiolate (MeS^–^) was employed as cysteine surrogate to evaluate the relative thermodynamic stability of the possible covalent adducts. The S–C bond between MeS^–^ and the corresponding electrophilic carbon atom in OTT/WIT was manually generated using PyMOL v2.5,^50^ followed by an initial geometry optimization of the adducts at the GFN2-xTB^51^ level of theory using xTB v6.7.1.^52^ Conformational ensembles of each possible adduct and of the isolated reactants were generated with CREST v3.0.2 ^53^ at the previous semiempirical level of theory. The resulting global minimum-energy conformations were then optimized at the SMD­(water)-M06-2X/def2-TZVP level of theory employing Gaussian v16. Vibrational frequency analyses at the same level of theory were carried out to obtain the corresponding thermal corrections to the electronic energy at 298.15 K and 1 bar. Reaction Gibbs free energies were calculated as Δ*G*_r_ = *G*_adduct_ – (*G*_OTT/WIT_ + *G*_MeS–_), using the fully optimized and infinitely separated reactants as the reference state. A standard-state correction of −1.89 kcal/mol was applied to the bimolecular association reactions to convert the calculated values from 1 bar to 1 mol/L.^54^

### Protein Preparation

A total of 138 tubulin crystal structures containing ligands bound to the COLbs were retrieved from the PDB^55^ (Table S1 of the Supporting Information). Using UCSF Chimera v1.16,^56^ each crystal structure was prepared by isolating the A and B chains, followed by the removal of the C, D, and E chains, as well as solvent molecules, cofactors, and ions. Missing loop regions were modeled using Modeller v10.8 ^57^ to generate complete structural models.

### Ligand Preparation

The three-dimensional structures of OTT and WIT were obtained from the PubChem database.^58^ Covalent complexes of each ligand with a capped cysteine residue (acetyl group [ACE] + cysteine [CYS] + *N*-methylamide [NME]) were then constructed, generating ACE-[CYS-OTT/WIT]-NME adducts. For OTT, all five adducts arising from 1,6- and 1,4-Michael addition reactions were generated, whereas for WIT, both the (*R*)*-* and (*S*)*-*enantiomers resulting from the 1,4-Michael addition reaction were considered. Based on crystallographic data, the phi (ϕ) and psi (ψ) dihedral angles of the cysteine backbone were set to −67.6 and −20.8°, respectively, while the amide dihedral angles were fixed at 180°, corresponding to the *trans* isomer. Subsequently, a simulated annealing algorithm was employed to generate multiple conformations of each adduct. All generated conformations obtained were subjected to semiempirical energy minimization using the PM6 method^59^ as implemented in Gaussian v16. The 20 lowest-energy conformations were then subjected to geometry optimization using the B3LYP functional and the 6-31G­(d)^60^ basis set.

### Covalent Molecular Docking

We carried out an ensemble covalent docking of the OTT and WIT adduct enantiomers using the 138 tubulin crystal structures from the PDB. Covalent docking calculations followed the flexible side-chain protocol developed by Bianco et al.^61^ using AutoDock v4.2.6.^62^ Each optimized adduct was uncapped and superimposed onto the backbone atoms of the βCys239 residue in the corresponding tubulin models using PyMOL v2.5. A docking grid box of 30 × 30 × 30 Å^3^ with a grid spacing of 0.375 Å was defined and centered on βCys239. Docking parameters were set to 50 million energy evaluations, a population size of 150 individuals, and 100 independent runs. For OTT, pose selection was based on three criteria: the root-mean-square deviation (RMSD) relative to the 3′-hydroxyl-4′-methoxyphenyl moiety of CA-4 (PDB ID: 5LYJ^63^), the docking score, and cluster size. For WIT, pose selection was based on docking score values and the most representative binding orientations.

### Molecular Dynamics Simulations

A nonstandard residue parametrization strategy was employed to prepare the CYS-OTT and CYS-WIT covalent complexes for molecular dynamics (MD) simulations, employing GROMACS 2021.7 ^64^ and the AMBER14SB force field.^65^ The electrostatic potential (ESP) of each system was calculated using Gaussian v16 at the HF/6-31G­(d) level of theory with a tight self-consistent field (SCF) convergence criterion. Atomic charges were derived using the Merz–Kollman^66^ scheme and subsequently converted to restrained electrostatic potential (RESP) charges using *antechamber* (AmberTools 17 ^67^) and the built-in *resp* script. The RESP fitting followed a multistage protocol developed by Cornell et al.^68^ Partial charges for the cap atoms and the N, H, C, and O backbone atoms of the CYS-OTT/WIT residues were constrained to the corresponding AMBER14SB values; subsequently, all chemically equivalent atoms were also constrained and refitted to carry identical charges. The resulting nonstandard amino acid topology was translated using ACPYPE^69^ (v. 2022.7.21) and incorporated into the bonded and nonbonded force field files for use in GROMACS 2021.7. Finally, three independent MD replicas of 100 ns were performed on a capped tripeptide model, ACE-GLY-[CYS-OTT/WIT]-GLY-NME, in explicit water (∼17,000 molecules) and in a 40% (V/V) 2,2,2-trifluoroethanol (TFE)/water mixture (∼1,570 TFE molecules and ∼9,859 water molecules) to refine the parametrization (Figure S1 of the Supporting Information). The TFE/water mixture was employed to approximate the hydrophobic microenvironment of the COLbs. TFE parameters were taken from the fifth model reported by Gerig et al.^70^

Protein–ligand complexes obtained from the covalent docking study were subjected to three independent 200 ns unrestrained MD simulations using GROMACS 2021.7 with the modified AMBER14SB force field. Each system was solvated in a periodic cubic box with TIP3P water model and neutralized with 0.15 M NaCl. Following energy minimization, systems were equilibrated for 1.0 ns under canonical (NVT) and isothermal–isobaric (NPT) ensembles. A velocity-rescaling (V-rescale) thermostat^71^ was used to maintain the temperature at 300 K, while pressure was controlled at 1.0 bar using the Parrinello–Rahman barostat.^72^ A cutoff of 1.0 nm was applied for short-range electrostatic and Lennard-Jones interactions, and long-range electrostatics were treated using the Particle Mesh Ewald (PME)^73^ method. All covalent bonds involving hydrogen atoms were constrained using the LINCS algorithm.^74^ Trajectory analysis was performed on the final 100 ns of each MD replica. Protein backbone and ligand RMSD were calculated with respect to the protein backbone, and clustering analysis was performed based on ligand RMSD using a 0.15 nm cutoff with the built-in GROMACS tools. Dihedral angle distributions for the nonstandard amino acids (χ_1_ and χ_2_) were computed using the GROMACS *angle* utility. Hydrogen-bond and hydrophobic interaction fractions were determined using GROMACS and the Protein–Ligand Interaction Profiler (PLIP)^75^ v2.2.2, respectively.

Additionally, 1 μs MD simulations were performed for the apo form of the protein (PDB ID: 8CLC^76^) and for the covalent tubulin adducts formed by the two WIT isomers, using the same parameters applied in the 200 ns simulations. In these longer simulations, the distance between the center of geometry (COG) of residue Ala352 located in the β-S9 loop of β-tubulin and the COG of residues 244–248 forming the β-T7 loop of the same subunit was analyzed using MDAnalysis v2.7.^77^ Furthermore, the cavity volume based on the solvent-excluded surface (SES) of the COLbs was determined using pyKVFinder.^78^ For this analysis, the atom types and van der Waals radii of WIT were included in the input parameters. In addition, to ensure a consistent analysis region, all structures were aligned to the structure with PDB ID 7YHN^79^ and its cocrystallized ligand (Y48, ID: IUK), which occupies the three zones of the COLbs, thus serving as reference to define the cavity search region with a 5 Å cutoff around its structure. Herein, probe in and out values were set to 1.4 and 4.0 Å, respectively. The protein and ligand RMSD values, after alignment to the protein backbone, were also calculated, as well as the RMSF of the protein backbone.

Chemical structures were generated using ACD/ChemSketch v2021.2.0,^80^ plots were prepared with Gnuplot version 5.2,^81^ and three-dimensional representations of proteins and chemical structures were produced with PyMOL v2.5.

## Results and Discussion

### The Orbital-Weighted Dual Descriptor Reveals the Potential Reactive Atoms of OTT and WIT

Within conceptual DFT (CDFT), various approaches are available to evaluate a molecule’s reactivity toward a reactive species. Among them, the so-called “local descriptors” are the most widely accepted as these enable the characterization of individual reactive atoms, in contrast to “global descriptors” which provide a “whole-molecule” description.^82^ For electron-delocalized systems, the local Δ*f*_w_ by Pino-Rios and co-workers has shown remarkable reactivity predictions, where other methods (e.g., the Fukui functions or the regular dual descriptor) have performed worse.^46,83^ Hence, we chose this approach to describe both OTT and WIT, considering the α,β-unsaturated systems present in these molecules. Within this framework, electrophilic atoms are characterized by positive values of Δ*f*_w_ and nucleophilic atoms display negative values.

In the case of OTT, three electrophilic sites were identified: C3, C9, and C13 (Figure 2A). From the condensed-to-atom values of Δ*f*_w_ (Table S2 of the Supporting Information), the estimated order of electrophilicity is C3 > C9 > C13. Thus, OTT could potentially be involved in 1,2-, 1,4-, and 1,6-addition reactions when interacting with a nucleophile. Notably, its vinyl group (C22 and C23) would be unreactive toward such electron-rich species, as it is slightly nucleophilic (Figure 2A). In the case of WIT, two main electrophilic sites are observed: C7 and C11 with very similar condensed-to-atom values of Δ*f*_w_ (Table S3 of the Supporting Information). Therefore, WIT might participate in 1,2- and 1,4-addition reactions (Figure 2B). These reactivity patterns are consistent across the five functionals used, showing that the predicted reactive sites are robust and independent of the level of theory used.

**Figure 2:**
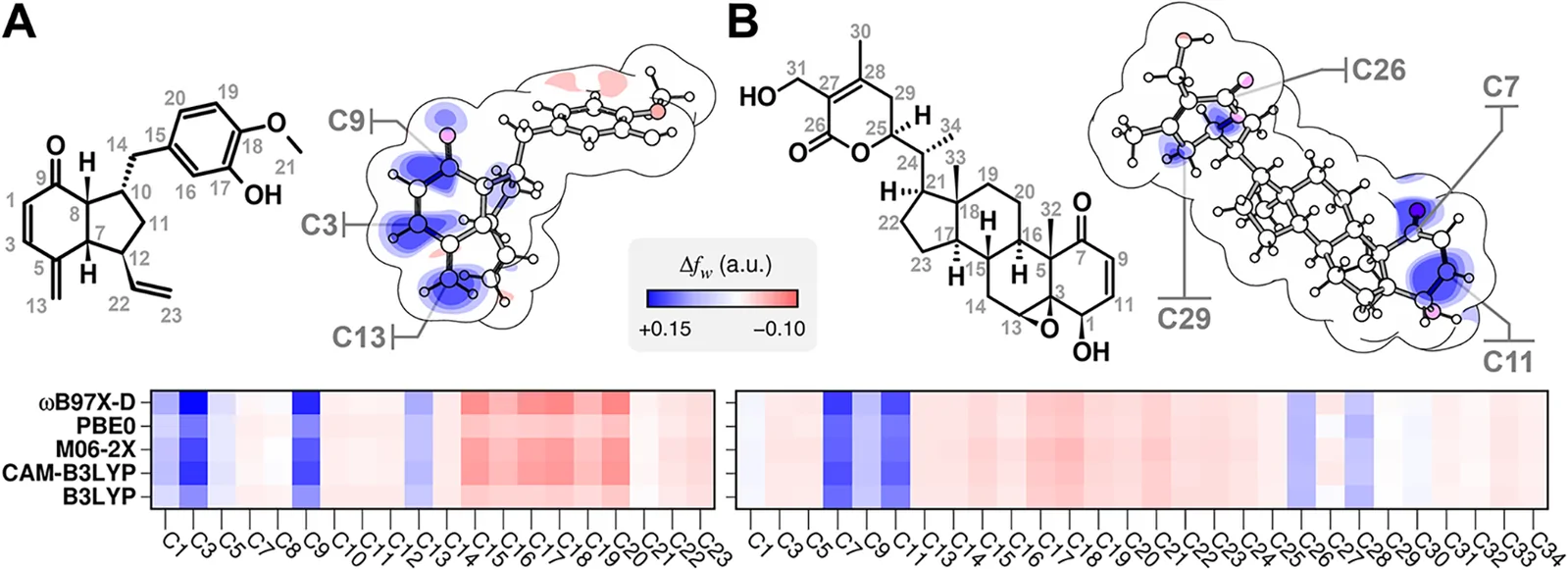
Regioselectivity analysis based on the orbital-weighted dual descriptor (Δ*f*_w_): (A) OTT, (B) WIT. The upper panels show the Δ*f*_w_ mapped onto the electron density isosurface (0.005 |*e*| Bohr^–3^) of the corresponding minimum-energy structures, computed at the SMD­(water)-PBE0-D3­(BJ)/def2-TZVP level of theory. Carbon atom IDs are shown in the 2D structures for reference and do not correspond to IUPAC atom numbering. To facilitate comparison across the five exchange-correlation functionals employed, the condensed-to-atom values of Δ*f*_w_ for each carbon atom are shown as heatmaps in the lower panels. Electrophilic sites (Δ*f*_w_ > 0) are shown in blue, whereas nucleophilic sites (Δ*f*_w_ < 0) are shown in red.

### Thermodynamic Analysis Favors 1,6-Addition in OTT and 1,4-Addition in WIT

To better understand the reactivity trends found by the previous CDFT-analyses for OTT and WIT, the relative thermodynamic stability of each possible covalent adduct was evaluated using methyl thiolate (MeS^–^) as cysteine surrogate. This approach has been extensively used to rationalize the reactivity profiles of diverse Michael acceptors targeting cysteines by comparing the Gibbs free energy of the resulting MeS-adducts in relation to the isolated reactants (Δ*G*_r_).^84–87^ To this end, the M06-2X density functional has shown remarkable accuracy, yielding theoretical Δ*G*_r_ values that closely resemble experimental data.^84,85^ Therefore, this level of theory was chosen for the corresponding thermochemical calculations.

For the MeS-OTT covalent adducts, a clear preference for 1,6-addition products is observed, with OTTa (Figure 1B) being the most stable adduct (Δ*G*_r_ = −7.52 kcal/mol), followed by OTTb (Δ*G*_r_ = −6.02 kcal/mol) and OTTc (Δ*G*_r_ = −5.85 kcal/mol). Although the latter adducts maintain an α,β-unsaturated system, the trisubstituted endocyclic alkene in OTTa maximizes hyperconjugative donation into the double bond, while also relieving localized steric strain.^88–90^ Such combination overrides the penalty of deconjugating the unsaturated dienone system. In contrast, formation of the 1,4-addition products is predicted to be only marginally favorable, being slightly exergonic for both OTTe (Δ*G*_r_ = −3.35 kcal/mol) and OTTd (Δ*G*_r_ = −1.61 kcal/mol). Their limited thermodynamic stability may arise from the formation of a sterically congested tetrahedral sp^3^ center within the six-membered ring, together with disruption of the conjugated α,β-unsaturated enone system.^88,91^ These results differ significantly from the 1,2-addition products, which result in highly endergonic adducts (Δ*G*_r_ > 7.08 kcal/mol). This pronounced endergonicity can be attributed to disruption of both the carbonyl π-bond and enone conjugation, coupled with the additional structural strain generated within the six-membered ring.^88,92^ Notably, this thermodynamic trend is also observed for the WIT adducts (Figure 1B), where 1,4-addition products WITa (Δ*G*_r_ = −5.69 kcal/mol) and WITb (Δ*G*_r_ = −6.78 kcal/mol) are more favorable than those resulting from the 1,2-addition reaction (Δ*G*_r_ > 11.25 kcal/mol). Overall, these results demonstrate that while CDFT local descriptors aid in screening reactive sites, intrinsic regioselectivity is ultimately governed by a combination of electronic and steric factors that these isolated descriptors cannot fully capture.

### OTT Occupies Z1 and Z2 Regions of the COLbs

OTT is a NP for which experimental studies have suggested its binding to the COLbs of β-tubulin, potentially involving a thiol-addition reaction with βCys239.^17^ To identify the reactive site and enhance the potency of OTT, Shaa’s group developed and evaluated a series of OTT analogs for antiproliferative activity and inhibition of microtubule polymerization.^18^ In that work, the authors demonstrated that the vinyl group (C22 and C23 in Figure 2A) did not significantly affect antiproliferative activity against several cancer cell lines, which is also consistent with our QM calculations. However, the analog lacking both the vinyl group and the exocyclic methylidene group (C5 and C13 in Figure 2A) showed a complete loss of activity across all six tested cancer cell lines. This finding suggested that structural simplification is possible by removing the vinyl group, whereas the exocyclic methylidene substituent, which enables 1,6-addition, is essential for antiproliferative activity. Moreover, the study proposed that the 3′-hydroxyl-4′-methoxyphenyl substituent of OTT occupies Z1 region within the COLbs, similarly to CA-4.

Since our QM calculations indicated that both 1,6- and 1,4-Michael additions are thermodynamically feasible, we performed an ensemble covalent docking protocol to evaluate the resulting covalent complexes. Five adducts were generated: three from 1,6-additions (OTTa, OTTb, and OTTc) and two from 1,4-additions (OTTd and OTTe), formed by covalent attachment of βCys239 to OTT (Figure 1B), and evaluated against 138 αβ-tubulin heterodimer structures. To identify the most plausible binding poses, AutoDock calculated scores were analyzed together with RMSD values computed relative to the geometric center of the aromatic ring and the methoxy oxygen of the 3′-hydroxy-4′-methoxyphenyl ring of CA-4 (PDB ID: 5LYJ^63^) (Figure 3). Several binding modes were identified for the three possible 1,6-adducts, some of which exhibited docking scores below −8.0 kcal/mol, RMSD values below 2.0 Å, and cluster populations greater than 75%. A total of 39 poses meeting the aforementioned docking score and RMSD criteria were obtained for OTTa, 11 for OTTb, and 26 for OTTc, suggesting that OTTa and OTTc are the most likely adducts formed during tubulin binding of OTT. Furthermore, docking results supported the earlier proposal that the 3′-hydroxyl-4′-methoxyphenyl substituent of OTT occupies the same space in region Z1 of the COLbs as CA-4. In contrast, very few poses were obtained for the two adducts from the 1,4-addition, consistent with Shaa’s observations (Figure S2 of the Supporting Information). Specifically, 11 poses for OTTd and only one pose for OTTe met the criteria. Interestingly, although the docking results were often associated with highly populated clusters, many exhibited either high RMSD values or less favorable scores. These findings suggest that, although the corresponding electrophilic group may exhibit suitable reactivity toward 1,4-addition, the resulting adducts may be less likely to adopt favorable binding orientations within the COLbs.

**Figure 3:**
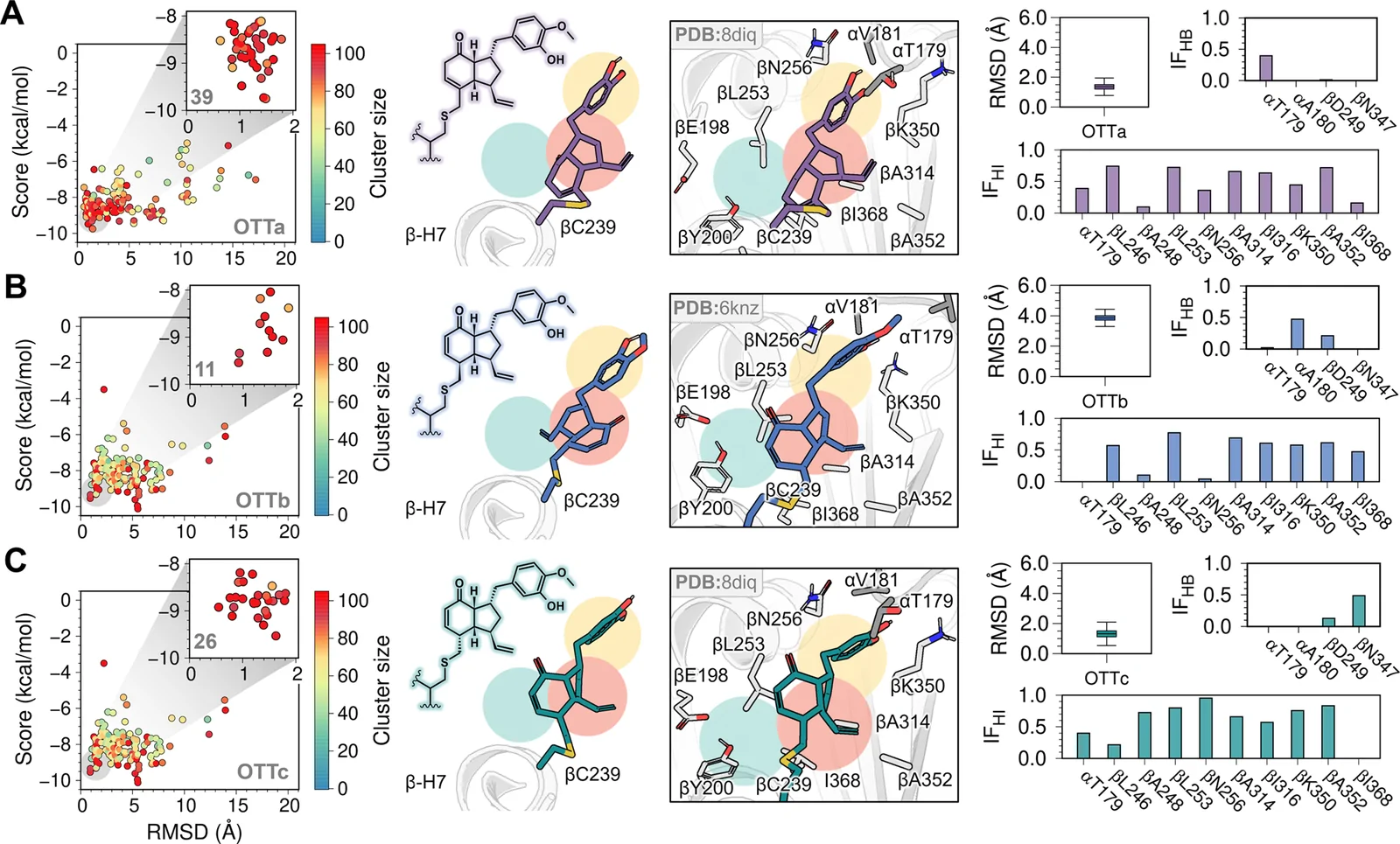
Molecular docking and molecular dynamics analysis of αβ-tubulin covalent adducts formed through 1,6-Michael addition of OTT: (A) OTTa, (B) OTTb, and (C) OTTc. Plots on the left show the docking score obtained with AutoDock and the RMSD of the 3′-hydroxyl-4′-methoxyphenyl moiety relative to the same substituent of CA-4 in the PDB structure 5LYJ. A zoomed-in view of the region of the plot corresponding to the most favorable docking scores (<−8.0 kcal/mol) and the lowest RMSD values (<2.0 Å) is shown to facilitate visualization. The center-left panel displays the chemical structure of the evaluated adduct and its docking pose relative to regions Z1 (yellow), Z2 (orange), and Z3 (blue) of the COLbs. The center-right panel shows the most representative ligand conformation obtained from the combined analysis of the last 100 ns of the three independent MD simulations performed for each complex. Plots on the right present the average ligand RMSD values and the interaction fractions (IF) for hydrogen bonds (HB) and hydrophobic interactions (HI) with the α- and β-tubulin subunits. Boxplots show the median, interquartile range (Q1–Q3), and whiskers extending to 1.5 × IQR (outliers are omitted for clarity).

Once the most probable OTT adducts in the αβ-tubulin heterodimer were identified, we performed three independent 200 ns MD simulations for each covalent tubulin-OTT complex to provide a more comprehensive characterization of OTT within the COLbs (Figure S3 of the Supporting Information). These MD simulations showed that OTTa and OTTc, which also yielded the highest number of favorable docking poses, maintained an average RMSD below 2.0 Å during the final 100 ns of all three replicas, preserving conformations highly consistent with those obtained from docking. By contrast, OTTb underwent a conformational shift, displacing the 3′-hydroxyl-4′-methoxyphenyl substituent from Z1. Across all three adducts, the interaction fraction of hydrogen bonds (IF_HB_) was variable, involving different residues, mainly formed with amino acids comprising Z1 of the COLbs and the α-T5 loop of the α-tubulin subunit. This is expected, since this site preferentially recognizes hydrophobic groups, as previously observed for other microtubule polymerization inhibitors occupying the COLbs.

In line with this, hydrophobic interactions of OTT adducts were found with several residues from both Z1 and Z2 regions. Among them, βAsn256 and βLys350 are key contributors to stabilizing inhibitors at Z1, and such interactions were evident for OTTa and OTTc, though less pronounced for OTTb. Between the former two, OTTc exhibited a greater fraction of hydrophobic interactions (IF_HI_) involving the 3′-hydroxyl-4′-methoxyphenyl substituent within Z1, suggesting a stronger preference for this adduct in the covalent complex. Additionally, interactions with αThr179 were particularly noteworthy, as compounds binding at the COLbs are known to interact either directly or indirectly (via water bridges) with this residue. Meanwhile, in Z2, residues βLeu253, βAla314, βIle316, and βAla352, well established as contributors to hydrophobic ligand interactions at the COLbs, showed high interaction prevalence during simulations, again most prominently for OTTa and OTTc. Moreover, βLeu246 and βAla248, which form part of the β-T7 loop, interacted with the cyclohexanone ring. Overall, OTTa and OTTc appear to display binding modes and interaction profiles most consistent with those reported in the literature for CA-4 and other tubulin polymerization inhibitors.

### WIT Can Adopt Two Main Conformations within the COLbs

WIT is a NP whose ergostane core confers a distinctive structural framework for binding to the COLbs, as no closely related scaffolds are present in the PDB, unlike the case of OTT. Nevertheless, experimental studies have demonstrated that this molecule, as well as other steroidal compounds (e.g., 2-methoxyestradiol), can bind to this site in tubulin.^93^ For this molecule, our QM calculations indicate that 1,4-Michael addition products are thermodynamically favored. Accordingly, the binding mode analysis of WIT is focused on the corresponding 1,4-covalent adducts.

In contrast to the covalent docking results obtained for OTT, only 38 and 3 poses with docking scores close to −5.0 kcal/mol were obtained for the WITa and WITb adducts, respectively (Figure 4). Moreover, two main orientations of WIT within tubulin were identified for both isomers: one in which WIT occupies regions Z1 and Z2 of the COLbs (WIT-*in*), and another in which the molecule is oriented toward the αβ-tubulin intradimer interface (WIT-*out*). MD simulations revealed that the WIT-*in* conformation exhibits high stability within the binding site (RMSD ≈ 2 Å), forming several hydrophobic contacts with residues such as αVal181, βLeu253, βAsn256, βAla314, and βLys350, similarly to what was observed for OTT (Figures S4–S6 of the Supporting Information). A similar phenomenon has been reported in molecular dynamics simulations of 2-methoxyestradiol bound to the COLbs.^94^ In contrast, these interactions are substantially reduced in the WIT-*out* conformation, as the ligand remains largely exposed to the solvent. The lower number of interactions is also associated with the higher mobility of this conformation, with RMSD values exceeding 2 Å in both cases.

**Figure 4:**
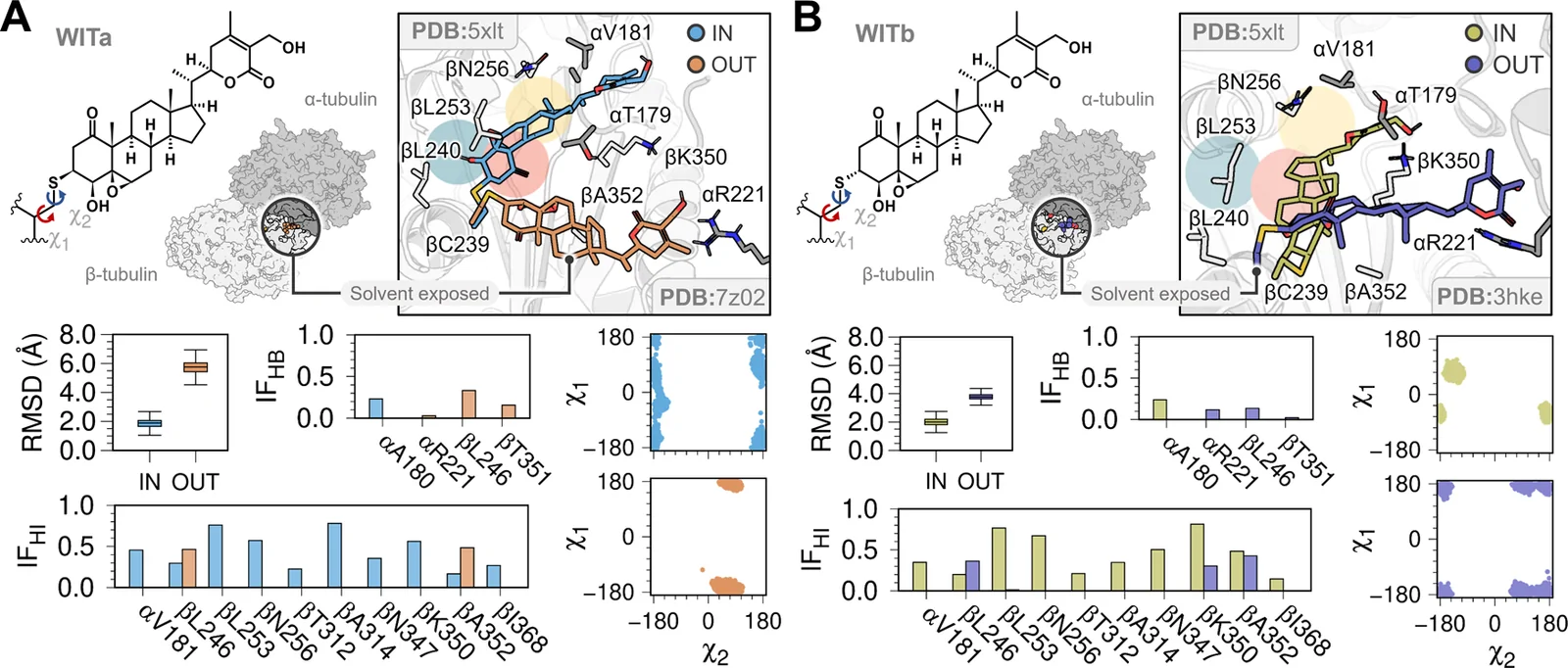
Molecular dynamics analysis of the two 1,4-Michael addition products of WIT in αβ-tubulin: (A) WITa and (B) WITb. For both adducts, the top panel shows the chemical structure with the χ_1_ (N-Cα-Cβ-Sγ) and χ_2_ (Cα-Cβ-Sγ-C11) dihedral angles indicated, along with the most representative structures of the two conformations (WIT-*in* and WIT-*out*) obtained from the last 100 ns of the three independent MD simulations performed for each complex. The bottom panels present the average ligand RMSD values and the interaction fractions (IF) for hydrogen bonds (HB) and hydrophobic interactions (HI) with the α- and β-tubulin subunits. Boxplots show the median, interquartile range (Q1–Q3), and whiskers extending to 1.5 × IQR (outliers are omitted for clarity). Additionally, the Janin plots of the χ_1_ and χ_2_ dihedral angles are shown.

Initially, we investigated whether this increased mobility could be related to rotation of the βCys239 side chain. However, analysis of the χ_1_ (N-Cα-Cβ-Sγ) and χ_2_ (Cα-Cβ-Sγ-C11) dihedral angles indicates that even the WIT-*in* conformation of WITa exhibits greater variability in the χ_1_ angle. Therefore, the increased mobility observed for the WIT-*out* conformations is primarily associated with the free rotation of the δ-lactone substituent. It is worth noting that in both WITa and WITb adducts, the number of hydrogen bond interactions is very low. Thus, as observed by most agents that bind to the COLbs, ligand binding appears to be predominantly mediated by hydrophobic interactions.

### Exposure of the COLbs by the WIT-*out* Conformation May Explain Tubulin Denaturation

Our molecular docking and MD simulations results suggest that the proposed 1,6- and 1,4-Michael addition adducts of OTT and WIT, respectively, adopt favorable binding conformations within the COLbs. However, in the case of WIT, we identified a binding pose in which the molecule, covalently linked to βCys239, is oriented toward the solvent at the αβ-tubulin heterodimer interface. Although at first glance this conformation might be considered incorrect or an artifact of the docking procedure, its occurrence may be related to the tubulin degradation mechanism reported for this molecule.

As mentioned previously, BAT is a synthetic compound that also forms a covalent bond with βCys239 of β-tubulin, and it has recently been shown to promote tubulin degradation through the intracellular ubiquitin-proteasome system, a mechanism that has also been reported for WIT.^23,29^ Unlike the two natural products examined in this work, BAT has been cocrystallized with tubulin (PDB ID: 3HKE).^30^ Although this structure was solved at relatively low resolution (3.6 Å), it revealed the presence of two BAT molecules in the COLbs: one bound noncovalently within regions Z1 and Z2, and a second molecule covalently bound to βCys239, oriented toward the αβ-tubulin heterodimer interface (BAT-*out*).

Although the covalent binding mechanism of BAT has not yet been fully explored, studies of covalent ligands targeting the peroxisome proliferator-activated receptor γ (PPARγ) have shown that the binding of covalent compounds may involve distinct precovalent and postcovalent binding poses, with covalent bond formation promoting a rearrangement of the ligand within the binding site.^95^ Accordingly, it is plausible that BAT, as well as WIT, initially adopts a precovalent pose, involving reversible-binding mode within the COLbs. This orientation would position the electrophilic reactive group (“warhead”) toward the sulfur atom of βCys239. Once the covalent bond is formed, the ligand could rearrange to adopt a postcovalent state, in which the molecule reorients toward the heterodimer interface, promoting displacement of the β-T7 loop. In the tubulin-BAT complex, the BAT-*out* orientation may result from such a postcovalent rearrangement or may have been promoted by competitive binding of a second BAT molecule. Regardless of the exact mechanism, the WIT-*out* orientation identified in this work closely resembles the BAT-*out* conformation determined experimentally (Figure 5A).

**Figure 5:**
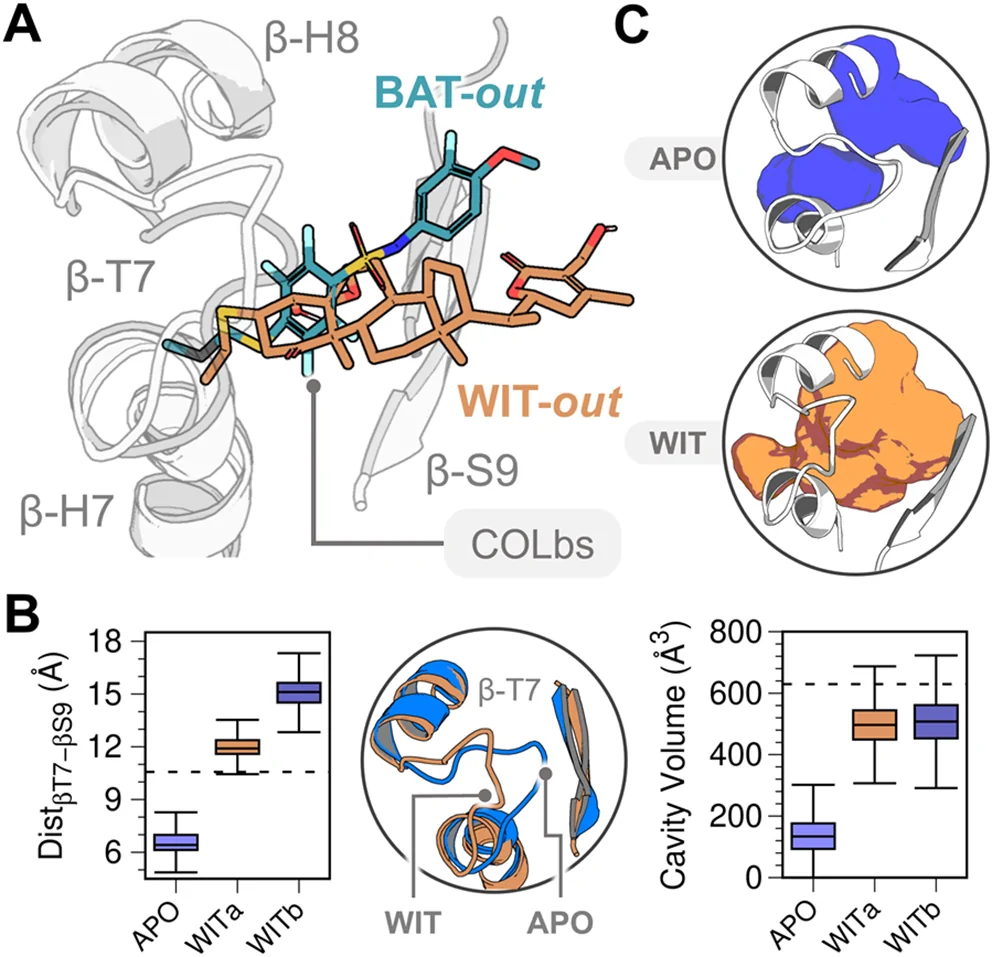
(A) Comparison of the interface-oriented conformations of BAT (BAT-*out*, teal) and WITa (WIT-*out*, orange) in the αβ-tubulin heterodimer. (B) Distance between the β-T7 loop and βAla352 of the β-S9 strand (Dist_βT7‑βS9_) measured during the 1 μs MD simulations of the system in the apo form and in complex with the two WIT adducts (WITa and WITb). The right panel provides a visual representation of the conformational differences of the β-T7 loop between the systems. (C) Analysis of the COLbs cavity volume (Å^3^) during the 1 μs MD simulations of the apo αβ-tubulin system and of the complexes with WITa and WITb. The top panels visually illustrate the differences in cavity size among the systems. The dotted lines in panels (B) and (C) represent experimental values derived from the crystallographic structure of the tubulin-BAT complex (PDB ID: 3HKE). Boxplots show the median, interquartile range (Q1–Q3), and whiskers extending to 1.5 × IQR (outliers are omitted for clarity).

To investigate whether the WIT-*out* conformation could contribute to tubulin destabilization, potentially explaining the effect also observed for BAT, we extended one replica of both WITa and WITb adducts in this orientation to 1 μs MD simulations, and compared them with simulations of the apo αβ-tubulin heterodimer (Figure S7 of the Supporting Information). Analysis of the protein RMSD and RMSF did not reveal significant global structural changes, suggesting that the overall fold of the protein remains stable during the simulation time scale. However, protein denaturation processes may require nonconventional MD approaches, longer simulation times, or altered temperature conditions to observe structural disruption. Since our goal was to identify early structural events that might initiate denaturation, rather than to simulate the entire unfolding process, we focused our analysis on local changes within the binding site.

Analysis of the COLbs revealed two notable structural changes that could contribute to the initiation of tubulin destabilization by these compounds. First, we examined the displacement of the β-T7 loop by measuring the minimum distance between this loop and βAla352, a residue located in the β-S9 strand that interacts with β-T7 when the loop adopts its closed conformation (Dist_βT7‑βS9_, Figure 5B). In the apo form, Dist_βT7‑βS9_ parameter remains below 7.0 Å for most of the simulation time. In contrast, in both WIT-*out* simulations, the distance exceeds 11.0 Å for most of the trajectory, consistent with the BAT crystal structure, where a Dist_βT7‑βS9_ of 10.6 Å is observed. This outward displacement of the β-T7 loop allows the ligand to reorient toward the heterodimer interface. Once the ligand adopts this orientation, the COLbs becomes fully exposed to the solvent.

Because most residues forming the COLbs pocket are hydrophobic, water penetration into the cavity could destabilize the β-tubulin core. It is well-known that the exposure of hydrophobic cavities in proteins can induce their denaturation.^96^ To evaluate structural changes in the binding site, we analyzed the cavity volume of the COLbs (Figure 5C). This analysis showed that in the apo structure the pocket remains mostly closed (<200 Å^3^). In contrast, when WIT or BAT adopts the interface-oriented conformation, the cavity volume increases to over 400 Å^3^. The resulting increase in the solvent exposure of hydrophobic residues at the intradimer interface may promote tubulin destabilization and local unfolding, as previously described for other MDgAs.^31,97^ These structural alterations could ultimately lead to tubulin denaturation and aggregation, followed by ubiquitination and subsequent degradation. Notably, hydrophobic residues at the tubulin intradimer interface play an important role in monomer–monomer interactions and have been associated with the intrinsic instability of isolated tubulin monomers.^98^ Thus, increased solvent exposure of these residues could potentially contribute not only to local conformational perturbations but also to destabilization of the αβ-tubulin heterodimer.

The mechanism proposed herein may be particularly relevant to βCys239, given its unique location within the central region of the intradimer interface (Figure S8 of the Supporting Information). In contrast, covalent modification of other cysteine residues may have distinct structural and functional consequences depending on their location within the tubulin structure. For instance, covalent modification of βCys303, located near the protofilament interface, could potentially perturb interactions between protofilaments without necessarily affecting the intrinsic stability of the αβ-tubulin heterodimer.^23,24^ Similarly, covalent modification of cysteine residues in α-tubulin located at the interdimer interface would be expected to primarily interfere with interactions between adjacent tubulin heterodimers, thereby promoting microtubule depolymerization. An example of this behavior was recently reported for the oxidated form ofcacalol, an NP that covalently binds to αCys347 at the interdimer interface and promotes microtubule depolymerization by disrupting interactions between adjacent tubulin heterodimers.^99^

It should be emphasized, however, that the exposure of hydrophobic residues and subsequent destabilization resulting from covalent modification of βCys239 represents only one potential mechanism underlying the observed effects, as this residue may have broader roles in tubulin stability and function. Indeed, previous studies have suggested that interactions with βCys239, including the formation of a weak hydrogen bond, may contribute to the binding and activity of certain colchicine-site ligands.^100–102^ Beyond its potential role in ligand recognition at the COLbs, recent findings suggest that residue 239 in the βIII-tubulin isotype may allosterically modulate the affinity of paclitaxel, a microtubule-stabilizing agent.^103^ Evaluation of the βIII^S239C^-tubulin mutant revealed increased drug efficacy, which was attributed to enhanced paclitaxel binding affinity. Structural analyses further demonstrated that perturbation of residue 239 induces long-range conformational changes affecting the taxane-binding pocket, intertubulin interactions, and the nucleotide-binding site. These observations raise the possibility that covalent modification of βCys239 may similarly perturb structural and catalytic properties of tubulin, thereby contributing to the observed degradation phenotype through mechanisms beyond local destabilization of the intradimer interface.

Together, these results suggest a possible mechanism by which BAT and WIT may initiate tubulin destabilization, consistent with experimental observations. Furthermore, these findings support the possible existence of distinct precovalent and postcovalent binding states, as observed in other covalent ligand systems, which may be directly related to the tubulin-degrading activity of these and potentially other covalent MDgAs.

## Conclusions

In this work, we compiled previous experimental evidence on the covalent binding of OTT and WIT to βCys239 to characterize the resulting αβ-tubulin-ligand adducts and their potential mechanism of action. To this end, we first evaluated the intrinsic reactivity of the electrophilic warheads in both molecules using orbital-weighted dual descriptor analysis and thermodynamic calculations with MeS^–^ as cysteine surrogate. These analyses identified the most reactive atomic sites in OTT and WIT and clarified their potential reaction pathways toward nucleophiles. OTT displays three electrophilic centers, for which the most thermodynamically favorable covalent adducts arise from 1,6-addition reactions, whereas WIT presents two electrophilic sites of comparable reactivity, with 1,4-addition products being thermodynamically favored. Altogether, these results provide insight into the interplay of steric and electronic factors governing the covalent binding of these electrophilic NPs.

Molecular docking and MD simulations were then used to characterize the covalent binding modes of these ligands. The results suggest that OTT preferentially forms 1,6-Michael covalent adducts with βCys239 within the COLbs of β-tubulin, whereas WIT yields favorable 1,4-Michael adducts. Among the adducts evaluated, OTTa and OTTc exhibited the most favorable docking scores, stable binding poses, and persistent conformations during MD simulations, while OTT 1,4-addition adducts were poorly represented. These findings are consistent with experimental evidence indicating that the exocyclic methylidene group is essential for activity and confirm that the 3′-hydroxy-4′-methoxyphenyl substituent occupies the Z1 region of the COLbs similarly to CA-4.

For WIT, two principal binding orientations were observed: a stable WIT-*in* pose occupying the Z1 and Z2 regions of the COLbs and a more dynamic WIT-*out* conformation oriented toward the αβ-tubulin heterodimer interface. Although the WIT-*in* pose resembles the canonical binding mode of COL-site inhibitors, the WIT-*out* orientation parallels the experimentally observed BAT-*out* conformation and may represent a postcovalent binding state. Extended MD simulations indicate that this interface-oriented pose promotes displacement of the β-T7 loop and expansion of the COLbs cavity, increasing solvent exposure of the hydrophobic pocket. These structural changes may represent early events in tubulin destabilization and provide a mechanistic explanation for the tubulin-degrading activity reported for WIT, BAT, and related covalent ligands.

Overall, these results provide molecular-level insight into how these NPs interact with the COLbs of β-tubulin and suggest a plausible mechanism underlying their biological activity.

## Supplementary Material



## Data Availability

The files associated with the covalent docking and molecular dynamics simulations are freely available at: https://github.com/cadd111/CovNatProd.git.

## Notes

PDB codes of tubulin structures used in covalent molecular docking (Table S1); condensed-to-atom values of the orbital weighted dual descriptor for OTT (Table S2) and WIT (Table S3); molecular docking and molecular dynamics analyses of tripeptides (Figure S1), OTT (Figures S2 and S3) and WIT (Figures S4 and S5) adducts within αβ-tubulin, clustering analysis (Figure S6), 1 μs simulations of apo and tubulin-WIT systems (Figure S7), and tubulin heterotetramer highlighting the structural location of key cysteine residues (Figure S8) (PDF)
