# Supplementary material for: Elucidating the Covalent Binding of Ottelione A and Withaferin A at the Colchicine Site of β‑Tubulin
Source: ACS Omega. 2026 Aug 31;11(36):54196–207. doi: 10.1021/acsomega.6c05137 (PMC13584774; doi:10.1021/acsomega.6c05137)
Supplement: Supplementary file 1 [file ao6c05137_si_001.pdf]

Elucidating the Covalent Binding of Ottelione A and Withaferin A at the Colchicine Site of β-Tubulin

Mario Carreón-Escalante, Efrén Mar-Antonio, Samuel Sánchez-Maza, Hugo A. Salgado-Flores, and Rodrigo Aguayo-Ortiz*

Facultad de Química, Departamento de Farmacia, Universidad Nacional Autónoma de México, Mexico City 04510, Mexico
**Correspondence:* rodaguayo@comunidad.unam.mx

Table of contents

Contents		Page
**I. Supplementary Tables**		
**Table S1**	PDB codes of tubulin structures employed in the covalent molecular docking	S2
**Table S2**	Condensed-to-atoms values of the Orbital Weighted Dual Descriptor for OTT	S3
**Table S3**	Condensed-to-atoms values of the Orbital Weighted Dual Descriptor for WIT	S3
**II. Supplementary Figures**		
**Figure S1**	Molecular dynamics analysis of tripeptides	S4
**Figure S2**	Molecular docking analysis of OTT adducts obtained from the 1,4-Michael addition	S4
**Figure S3**	Molecular dynamics analysis of αβ-tubulin covalently bonded to OTTa, OTTb, and OTTc	S5
**Figure S4**	Molecular dynamics analysis of αβ-tubulin covalently bonded to WITa	S6
**Figure S5**	Molecular dynamics analysis of αβ-tubulin covalently bonded to WITb	S7
**Figure S6**	Percentage of structures represented by the three most populated clusters	S7
**Figure S7**	Molecular dynamics analysis of the 1 µs simulations of the apo and WIT αβ-tubulin systems	S8
**Figure S8**	Tubulin heterotetramer highlighting the structural location of key cysteine residues	S8
**III. Supplementary References**		S9

I. Supplementary Tables

Table S1. PDB codes of tubulin structures employed in the ensemble covalent docking study

PDB ID	Res. (Å)	Reference	PDB ID	Res. (Å)	Reference	PDB ID	Res. (Å)	Reference
1SA0	3.58	Ravelli *et al.*[1]	5XKE	2.60	Yan *et al.*[2]	6X1F	2.70	Banerjee *et al.*[3]
1SA1	4.20	Ravelli *et al.*[1]	5XKF	2.80	Yan *et al.*[2]	6XER	2.50	Chen, *et al.*[4]
3HKC	3.80	Dorléans *et al.*[5]	5XKG	2.20	Yan *et al.*[2]	6XES	2.32	Chen *et al.*[4]
3HKD	3.70	Dorléans *et al.*[5]	5XKH	2.25	Yan *et al.*[2]	6XET	2.60	Chen *et al.*[4]
3HKE	3.60	Dorléans *et al.*[5]	5XLT	2.81	Niu *et al.*[6]	6Y6D	2.20	Oliva *et al.*[7]
3N2G	4.00	Barbier *et al.*[8]	5XLZ	2.30	Cheng *et al.*[9]	7AU5	2.20	Yong *et al.*[10]
3N2K	4.00	Barbier *et al.*[8]	5YL2	2.09	Yang *et al.*[11]	7CBZ	2.61	Wang *et al.*[12]
4O2A	2.50	Prota *et al.*[13]	5YL4	2.64	Fu *et al.*[14]	7CDA	2.66	Yang *et al.*[15]
4O2B	2.30	Prota *et al.*[13]	5YLJ	2.70	Yang *et al.*[11]	7CE6	2.69	Yang *et al.*[15]
4YJ2	2.60	McNamara *et al.*[16]	5YLS	3.00	Yang *et al.*[11]	7CE8	2.73	Yang *et al.*[15]
4YJ3	3.75	McNamara *et al.*[16]	5YZ3	2.54	Ning *et al.*[17]	7CEK	2.70	Yang *et al.*[15]
5C8Y	2.59	Wang *et al.*[18]	5Z4P	2.50	Jiang *et al.*[19]	7CPD	2.51	Peng *et al.*[20]
5CA0	2.50	Wang *et al.*[18]	5Z4U	3.18	Lai *et al.*[21]	7CPQ	2.60	Peng *et al.*[20]
5CA1	2.40	Wang *et al.*[18]	5ZXH	2.80	Li *et al.*[22]	7DB9	2.85	Xian *et al.*[23]
5CB4	2.19	Wang *et al.*[18]	6AGK	2.80	Chen *et al.*[24]	7DBA	2.46	Ren *et al.*[25]
5GON	2.48	Zhou *et al.*[26]	6BR1	2.30	Banerjee *et al.*[27]	7DBB	2.81	Ma *et al.*[28]
5H7O	2.80	Arnst *et al.*[29]	6BRF	2.50	Banerjee *et al.*[27]	7DBC	2.40	Lei *et al.*[30]
5JCB	2.30	Zhao *et al.*[31]	6BRY	2.70	Banerjee *et al.*[27]	7DBD	3.09	Lei *et al.*[30]
5JVD	2.39	Canela *et al.*[32]	6BS2	2.65	Banerjee *et al.*[27]	7EMJ	2.33	Yan *et al.*[33]
5LP6	2.90	Marangon *et al.*[34]	6D88	2.85	Wang *et al.*[35]	7EXC	2.39	Li *et al.*[36]
5LYJ	2.40	Gaspari *et al.*[37]	6EG5	2.45	Arnst *et al.*[38]	7LZ7	2.80	Banerjee *et al.*[3]
5M7E	2.05	Bohnacker *et al.*[39]	6F7C	2.00	Cury *et al.*[40]	7LZ8	2.92	Banerjee *et al.*[3]
5M7G	2.25	Bohnacker *et al.*[39]	6FKJ	2.15	Bueno *et al.*[41]	7OGN	2.20	Oliva *et al.*[42]
5M8D	2.25	Bohnacker *et al.*[39]	6FKL	2.10	Bueno *et al.*[41]	7TTD	2.27	Pochampally *et al.*[43]
5M8G	2.15	Bohnacker *et al.*[39]	6GF3	2.40	Smedley *et al.*[44]	7TTE	2.70	Pochampally *et al.*[43]
5NFZ	2.10	Field *et al.*[45]	6GJ4	2.40	Brindisi *et al.*[46]	7TTF	2.10	Pochampally *et al.*[43]
5O7A	2.50	Sharma *et al.*[47]	6H9B	2.75	Naret *et al.*[48]	7VMG	2.39	Zhang *et al.*[49]
5OSK	2.11	Dohle *et al.*[50]	6HX8	2.40	Dohle *et al.*[51]	7VMJ	2.90	Zhang *et al.*[49]
5OV7	2.40	Jost *et al.*[52]	6JCJ	2.50	Zhang *et al.*[53]	7VMK	2.50	Zhang *et al.*[49]
5S4Q	2.59	Mühlethaler *et al.*[54]	6K9V	2.54	Li *et al.*[55]	7XQX	3.36	Tan *et al.*[56]
5S4R	2.35	Mühlethaler *et al.*[54]	6KNZ	2.48	Niu *et al.*[57]	7XQY	2.35	Tan *et al.*[56]
5S4S	2.35	Mühlethaler *et al.*[54]	6KPP	2.75	Wang *et al.*[58]	7XR0	2.70	Tan *et al.*[56]
5S4T	2.27	Mühlethaler *et al.*[54]	6LS4	2.40	Du *et al.*[59]	7XR1	2.81	Tan *et al.*[56]
5S4U	2.39	Mühlethaler *et al.*[54]	6LSM	2.75	Li *et al.*[60]	7YHN	2.60	Lin *et al.*[61]
5S4W	2.80	Mühlethaler *et al.*[54]	6LSN	2.44	Li *et al.*[60]	7YZ6	2.10	Wranik *et al.*[62]
5S4X	2.53	Mühlethaler *et al.*[54]	6N47	2.60	Arnst *et al.*[38]	7Z01	1.82	Gao *et al.*[63]
5S4Y	2.30	Mühlethaler *et al.*[54]	6NNG	2.40	Arnst *et al.*[64]	7Z02	2.36	Gao *et al.*[63]
5S4Z	2.10	Mühlethaler *et al.*[54]	6O5M	2.30	Wang *et al.*[65]	7Z2N	2.17	de la Roche *et al.*[66]
5S50	3.10	Mühlethaler *et al.*[54]	6O5N	3.00	Wang *et al.*[65]	7Z2P	2.00	de la Roche *et al.*[66]
5S51	2.40	Mühlethaler *et al.*[54]	6O61	2.60	Wang *et al.*[65]	7ZYW	2.45	Lucena-Agell *et al.*[67]
5S52	2.83	Mühlethaler *et al.*[54]	6PC4	2.60	Chen *et al.*[24]	8A9T	2.30	Barreca *et al.*[68]
5S53	2.75	Mühlethaler *et al.*[54]	6QQN	2.30	Patterson *et al.*[69]	8A9Z	2.29	Barreca *et al.*[68]
5XAF	2.55	Zhou *et al.*[70]	6TDE	2.29	Shchegravina *et al.*[71]	8C0F	2.10	Jernigan *et al.*[72]
5XAG	2.56	Zhou *et al.*[70]	6TH4	2.12	Stein *et al.*[73]	8CLB	3.00	Wranik *et al.*[74]
5XHC	2.75	Ding *et al.*[75]	6X1C	2.90	Banerjee *et al.*[3]	8CLF	2.70	Wranik *et al.*[74]
5XI7	2.99	Ding *et al.*[75]	6X1E	2.90	Banerjee *et al.*[3]	8DIQ	2.40	Deng *et al.*[76]

Table S2. Condensed-to-atom values of the Orbital Weighted Dual Descriptor for ottelione A (OTT)

Atom	DFT/def2-TZVP					Atom	DFT/def2-TZVP				
	B3LYP	CAM-B3LYP	M06-2X	PBE0	wB97XD		B3LYP	CAM-B3LYP	M06-2X	PBE0	wB97XD
C1	0.0319	0.0567	0.0540	0.0380	0.0705	C14	-0.0116	-0.0110	-0.0112	-0.0119	-0.0122
C3	0.0882	0.1267	0.1187	0.0974	0.1468	C15	-0.0302	-0.0570	-0.0542	-0.0337	-0.0651
C5	0.0166	0.0231	0.0193	0.0191	0.0287	C16	-0.0250	-0.0396	-0.0373	-0.0273	-0.0431
C7	-0.0096	-0.0051	-0.0072	-0.0088	-0.0048	C17	-0.0252	-0.0551	-0.0521	-0.0298	-0.0646
C8	-0.0067	0.0026	0.0012	-0.0055	0.0039	C18	-0.0281	-0.0596	-0.0566	-0.0328	-0.0698
C9	0.0851	0.1170	0.1106	0.0903	0.1318	C19	-0.0216	-0.0378	-0.0352	-0.0238	-0.0422
C10	-0.0127	-0.0084	-0.0098	-0.0123	-0.0085	C20	-0.0323	-0.0612	-0.0583	-0.0365	-0.0700
C11	-0.0116	-0.0064	-0.0071	-0.0111	-0.0059	C21	-0.0021	-0.0044	-0.0039	-0.0038	-0.0069
C12	-0.0118	-0.0072	-0.0088	-0.0110	-0.0063	C22	-0.0123	-0.0149	-0.0172	-0.0130	-0.0151
C13	0.0469	0.0595	0.0539	0.0532	0.0733	C23	-0.0179	-0.0202	-0.0227	-0.0182	-0.0198

Table S3. Condensed-to-atom values of the Orbital Weighted Dual Descriptor for withaferin A (WIT)

Atom	DFT/def2-TZVP					Atom	DFT/def2-TZVP				
	B3LYP	CAM-B3LYP	M06-2X	PBE0	wB97XD		B3LYP	CAM-B3LYP	M06-2X	PBE0	wB97XD
C1	0.0064	0.0087	0.0082	0.0059	0.0083	C21	-0.0270	-0.0271	-0.0311	-0.0267	-0.0262
C3	-0.0128	-0.0131	-0.0147	-0.0134	-0.0129	C22	-0.0191	-0.0172	-0.0199	-0.0189	-0.0165
C5	-0.0105	-0.0131	-0.0152	-0.0107	-0.0138	C23	-0.0197	-0.0191	-0.0219	-0.0199	-0.0187
C7	0.1026	0.1145	0.1061	0.1050	0.1242	C24	-0.0158	-0.0147	-0.0173	-0.0159	-0.0145
C9	0.0471	0.0514	0.0507	0.0497	0.0561	C25	-0.0080	-0.0083	-0.0092	-0.0084	-0.0087
C11	0.0926	0.1070	0.1010	0.0979	0.1162	C26	0.0606	0.0578	0.0616	0.0615	0.0583
C13	-0.0136	-0.0141	-0.0154	-0.0146	-0.0148	C27	0.0037	-0.0118	-0.0088	0.0039	-0.0131
C14	-0.0137	-0.0147	-0.0164	-0.0144	-0.0154	C28	0.0610	0.0482	0.0508	0.0632	0.0498
C15	-0.0199	-0.0219	-0.0250	-0.0200	-0.0218	C29	0.0004	-0.0014	-0.0018	0.0003	-0.0019
C16	-0.0127	-0.0144	-0.0176	-0.0125	-0.0135	C30	0.0081	0.0060	0.0059	0.0082	0.0053
C17	-0.0282	-0.0308	-0.0351	-0.0284	-0.0307	C31	-0.0060	-0.0081	-0.0069	-0.0074	-0.0088
C18	-0.0325	-0.0358	-0.0408	-0.0324	-0.0354	C32	-0.0046	-0.0033	-0.0046	-0.0049	-0.0036
C19	-0.0211	-0.0221	-0.0250	-0.0215	-0.0220	C33	-0.0133	-0.0121	-0.0139	-0.0135	-0.0119
C20	-0.0185	-0.0195	-0.0218	-0.0189	-0.0192	C34	-0.0094	-0.0076	-0.0091	-0.0096	-0.0076

II. Supplementary Figures

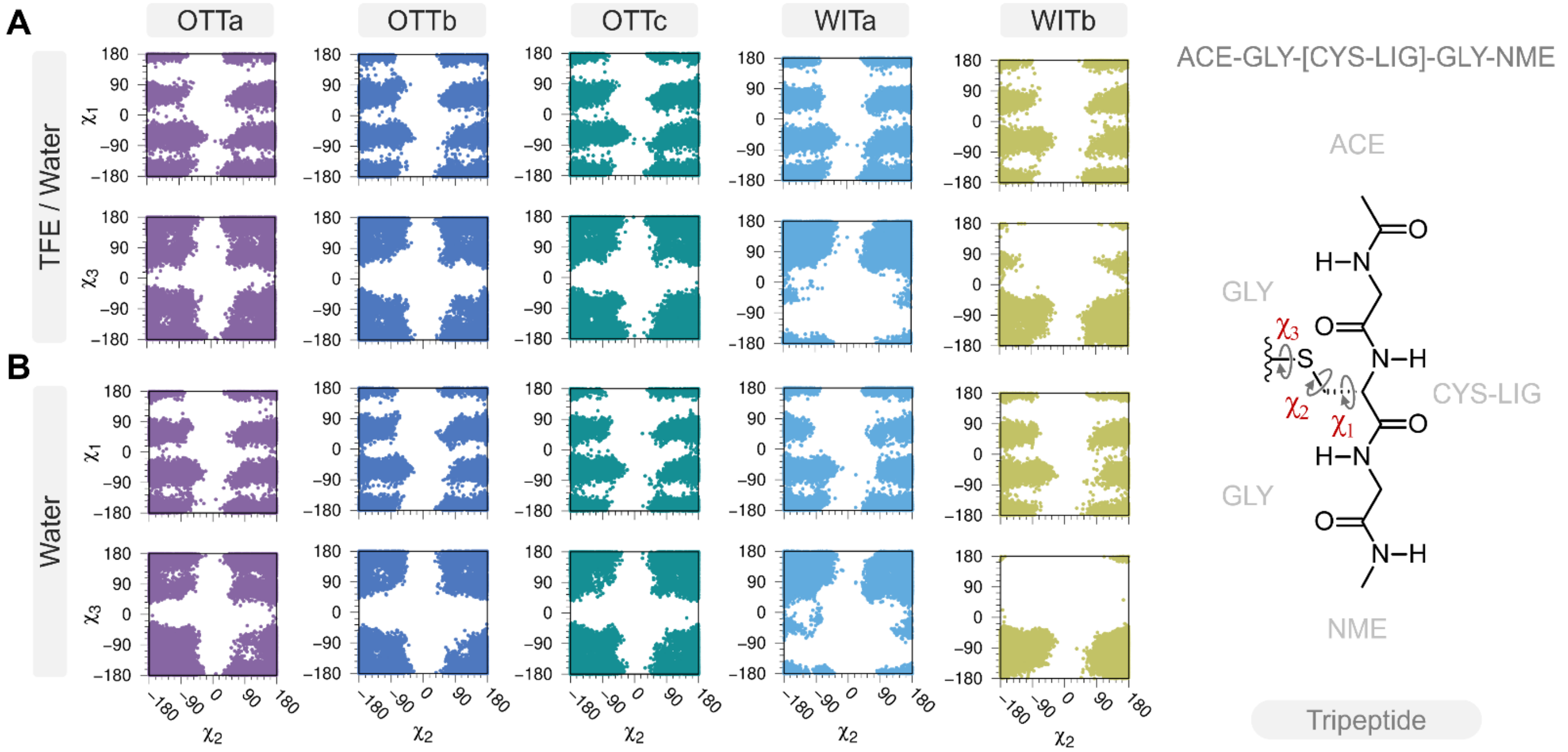

Figure S1. Analysis of the combined three independent 100 ns molecular dynamics replicas of tripeptides containing OTTa, OTTb, OTTc, WITa, and WITb, solvated in (A) a 40% (V/V) trifluoroethanol (TFE) mixture and (B) water. Janin plots show the χ_1, χ_2, and χ_3 dihedral angles for the five tripeptides. On the right, a representation of the capped tripeptides is shown, where the ligand (LIG) is covalently bonded to cysteine (acetyl group [ACE], glycine [GLY], cysteine + ligand [CYS-LIG], and *N*-methylamide [NME]).

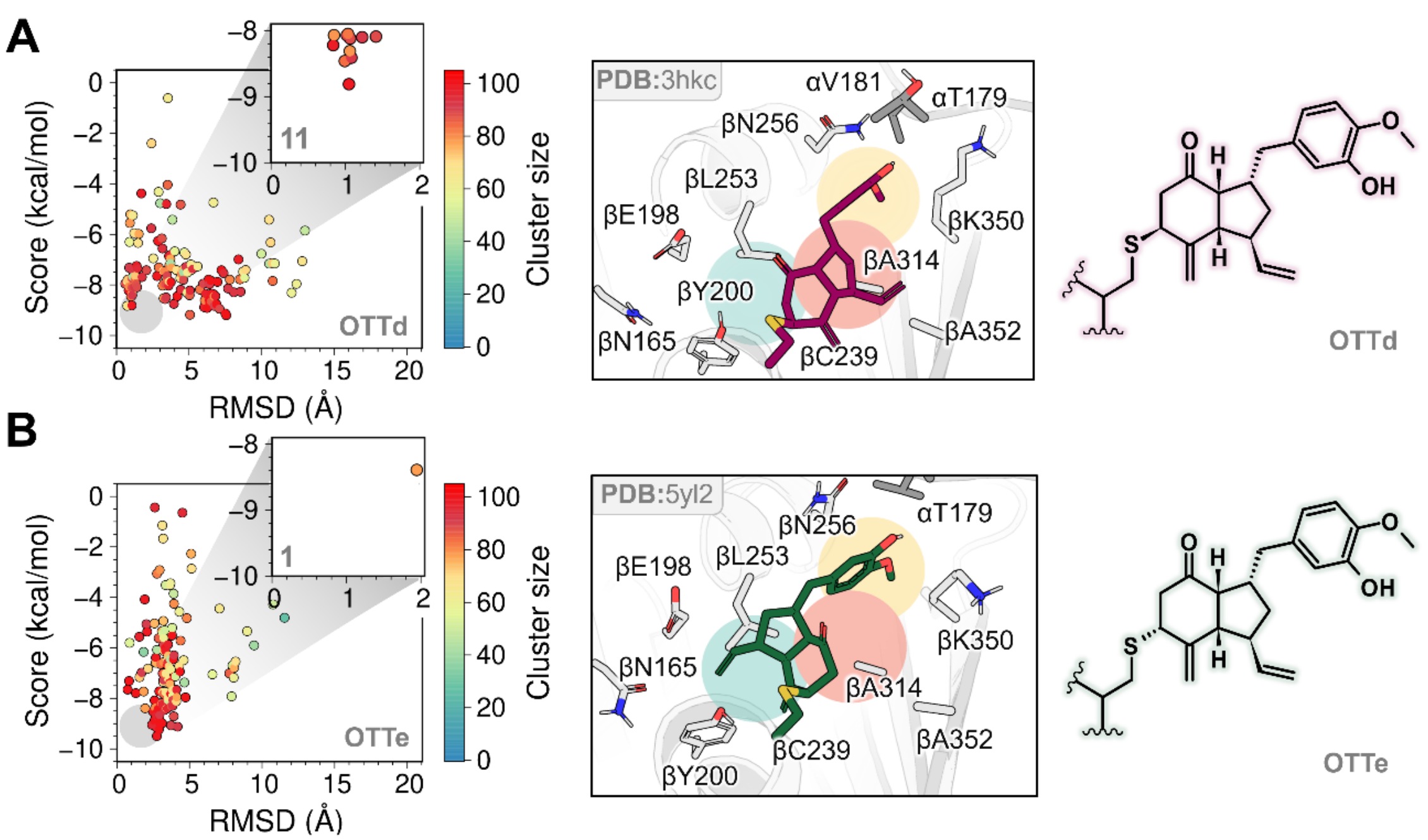

Figure S2. Molecular docking analysis of OTT adducts obtained from the 1,4-Michael addition: (A) OTTd and (B) OTTe. Plots on the left show the docking score obtained with AutoDock and the RMSD of the 3'-hydroxyl-4'-methoxyphenyl moiety relative to the same substituent of CA-4 in the PDB structure 5LYJ. A zoomed-in view of the region of the plot corresponding to the most favorable docking scores and the most similar RMSD values is shown to facilitate visualization. The center and right panels display the chemical structure of the evaluated adduct and its pose relative to regions Z1 (yellow), Z2 (orange), and Z3 (blue) of the COLbs.

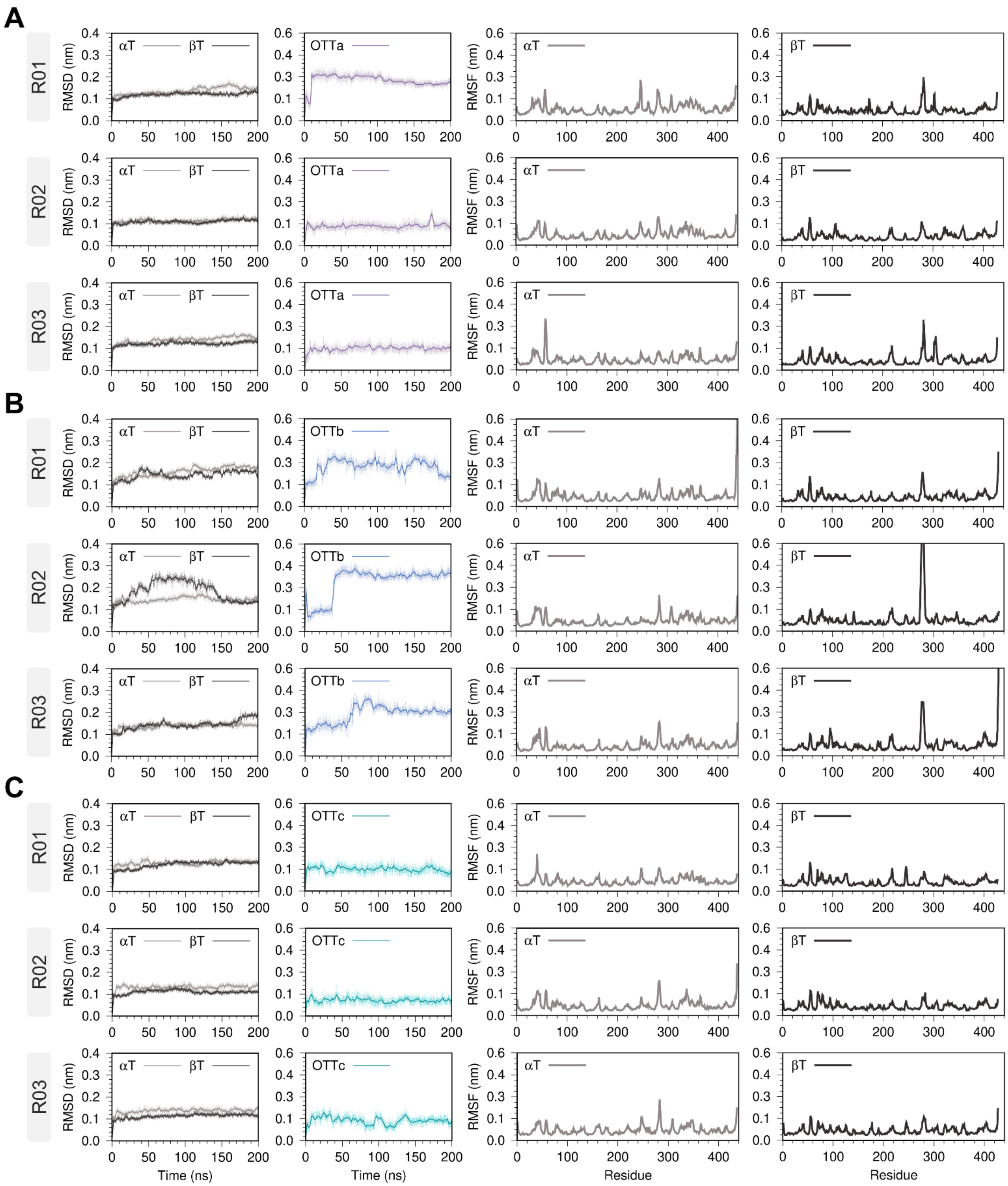

Figure S3. Molecular dynamics analysis of three independent 200 ns simulations (R01 to R03) of αβ-tubulin heterodimers containing the adducts (A) OTTa, (B) OTTb, and (C) OTTc. The leftmost plot shows the backbone RMSD of the α-tubulin (αT) and β-tubulin (βT) subunits throughout the simulation. The second plot from the left shows the RMSD of the ligand after least-squares fitting to the protein backbone. The two rightmost plots show the backbone RMSF of αT and βT subunits for the last 100 ns, respectively.

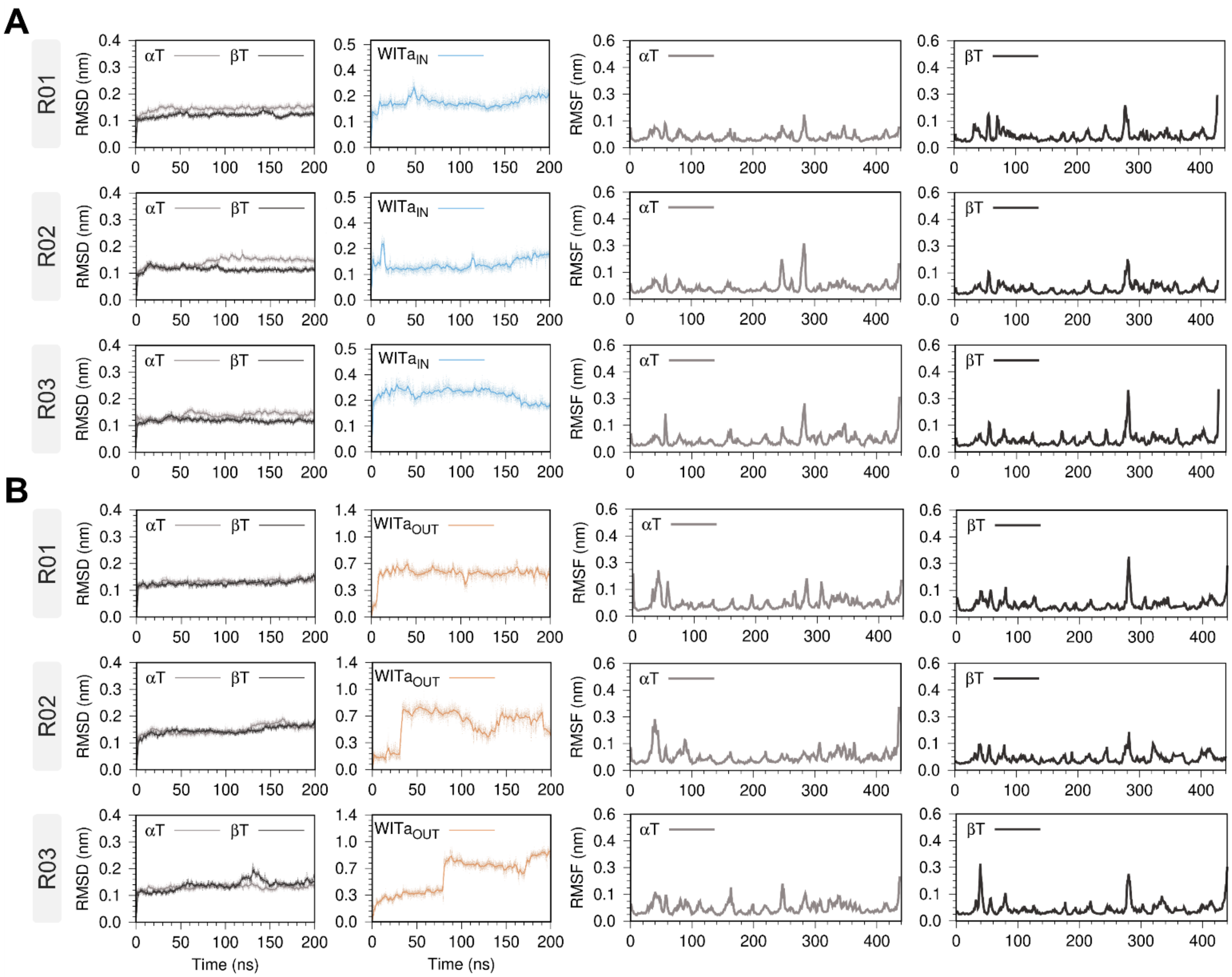

Figure S4. Molecular dynamics analysis of three independent 200 ns simulations (R01 to R03) of αβ-tubulin heterodimers containing the WITa adduct in the (A) WIT-*in* and (B) WIT-*out* conformations. The leftmost plot shows the backbone RMSD of the α-tubulin (αT) and β-tubulin (βT) subunits throughout the simulation. The second plot from the left shows the RMSD of the ligand after least-squares fitting to the protein backbone. The two rightmost plots show the backbone RMSF of αT and βT subunits for the last 100 ns, respectively.

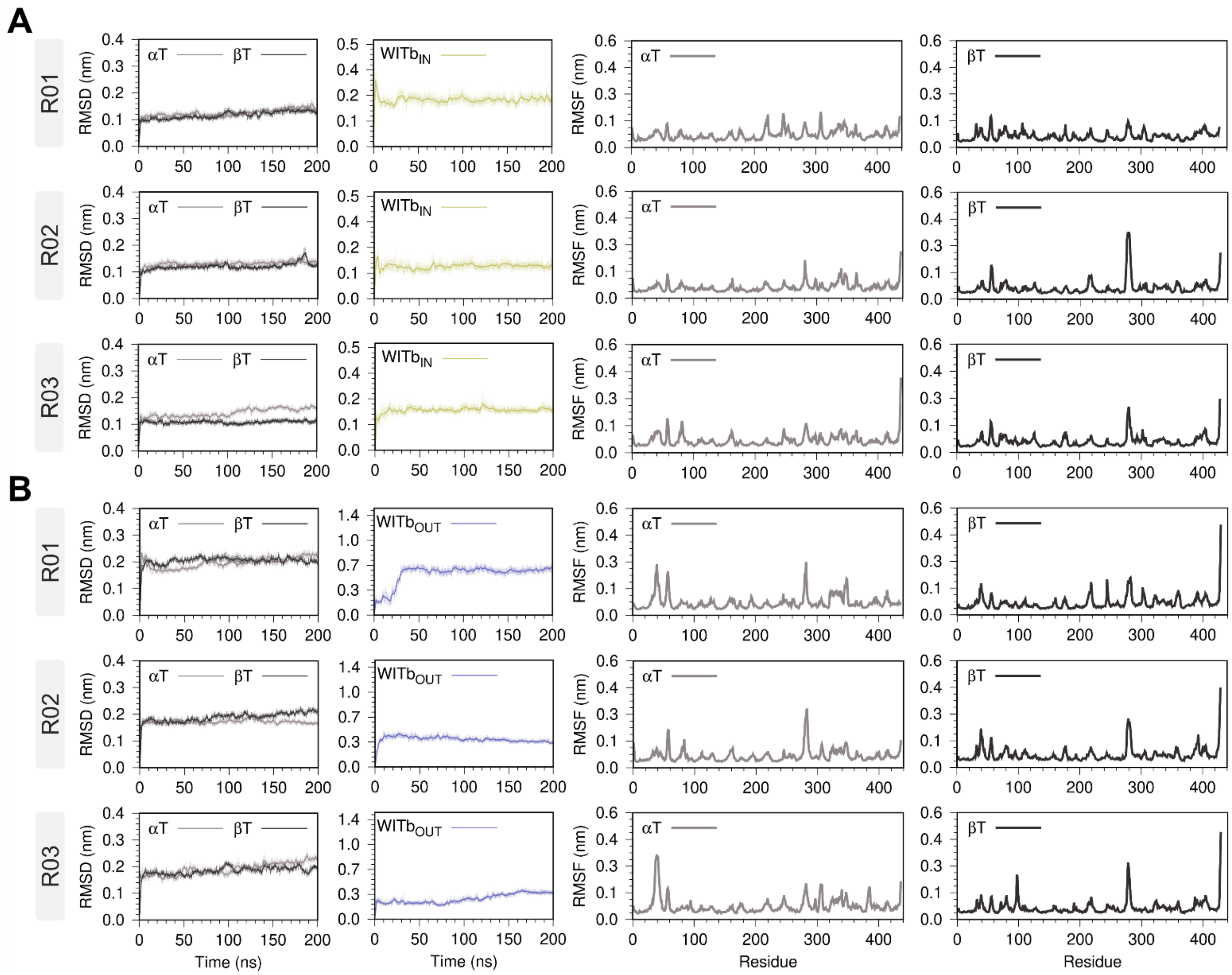

Figure S5. Molecular dynamics analysis of three independent 200 ns simulations (R01 to R03) of αβ-tubulin heterodimers containing the WITb adduct in the (A) WIT-*in* and (B) WIT-*out* conformations. The leftmost plot shows the backbone RMSD of the α-tubulin (αT) and β-tubulin (βT) subunits throughout the simulation. The second plot from the left shows the RMSD of the ligand after least-squares fitting to the protein backbone. The two rightmost plots show the backbone RMSF of αT and βT subunits for the last 100 ns, respectively.

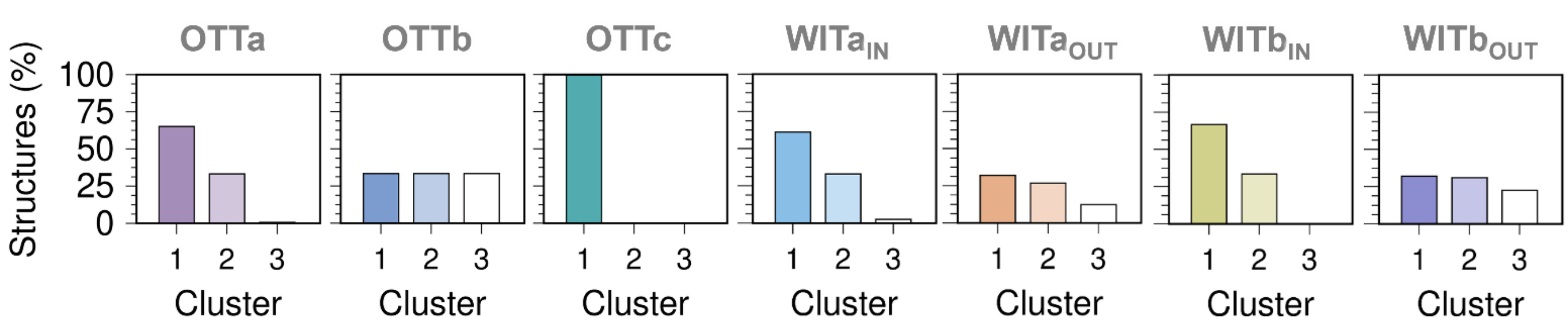

Figure S6. Percentage of structures represented by the three most populated clusters obtained from the combined analysis of the last 100 ns of the three independent molecular dynamics simulations from the seven studied systems.

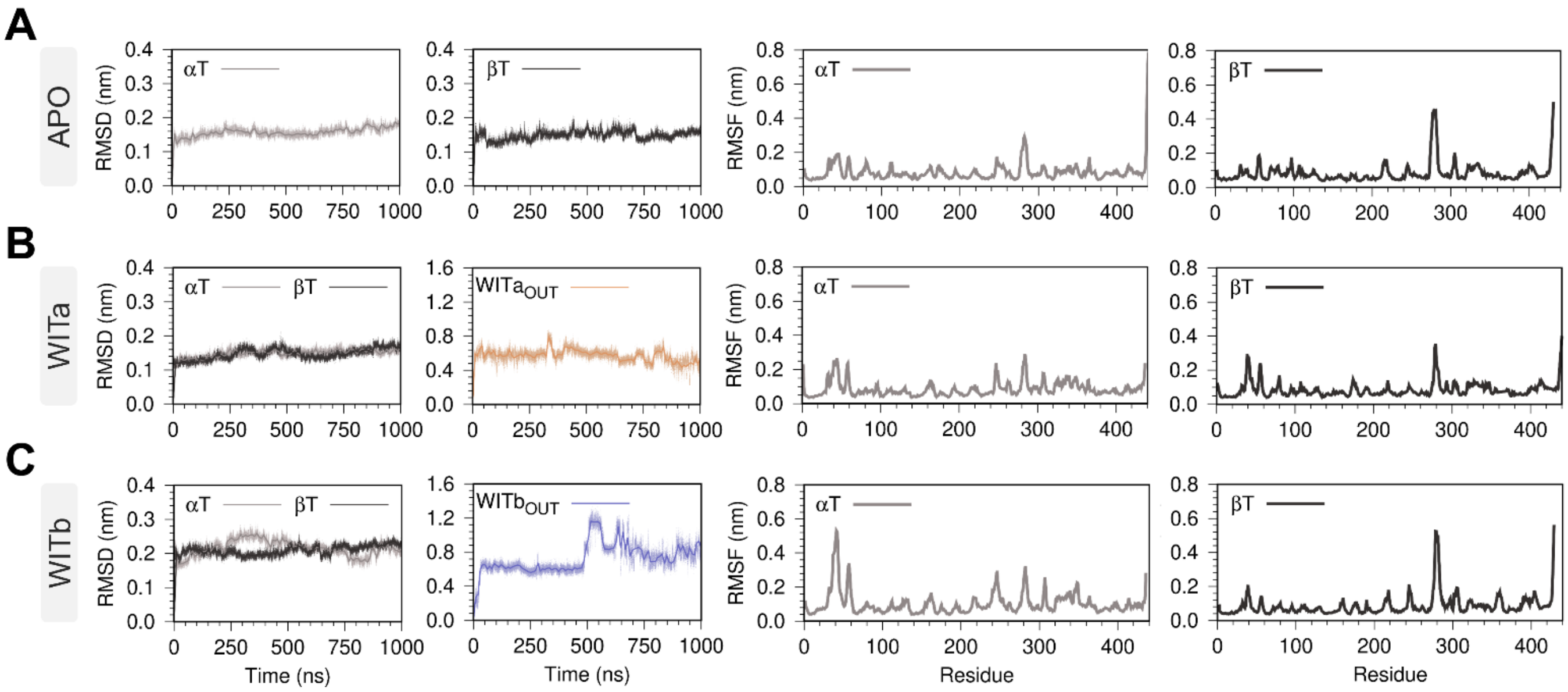

Figure S7. Molecular dynamics analysis of the 1 µs simulations of the (A) apo αβ-tubulin heterodimer and the systems containing the (B) WITa and (C) WITb adducts in the WIT-*out* conformations. The leftmost plot shows the backbone RMSD of the α-tubulin (αT) and β-tubulin (βT) subunits throughout the simulation. The second plots from the left of (B) and (C) show the RMSD of the ligand after least-squares fitting to the protein backbone. The two rightmost plots show the backbone RMSF of αT and βT subunits for the last 500 ns, respectively.

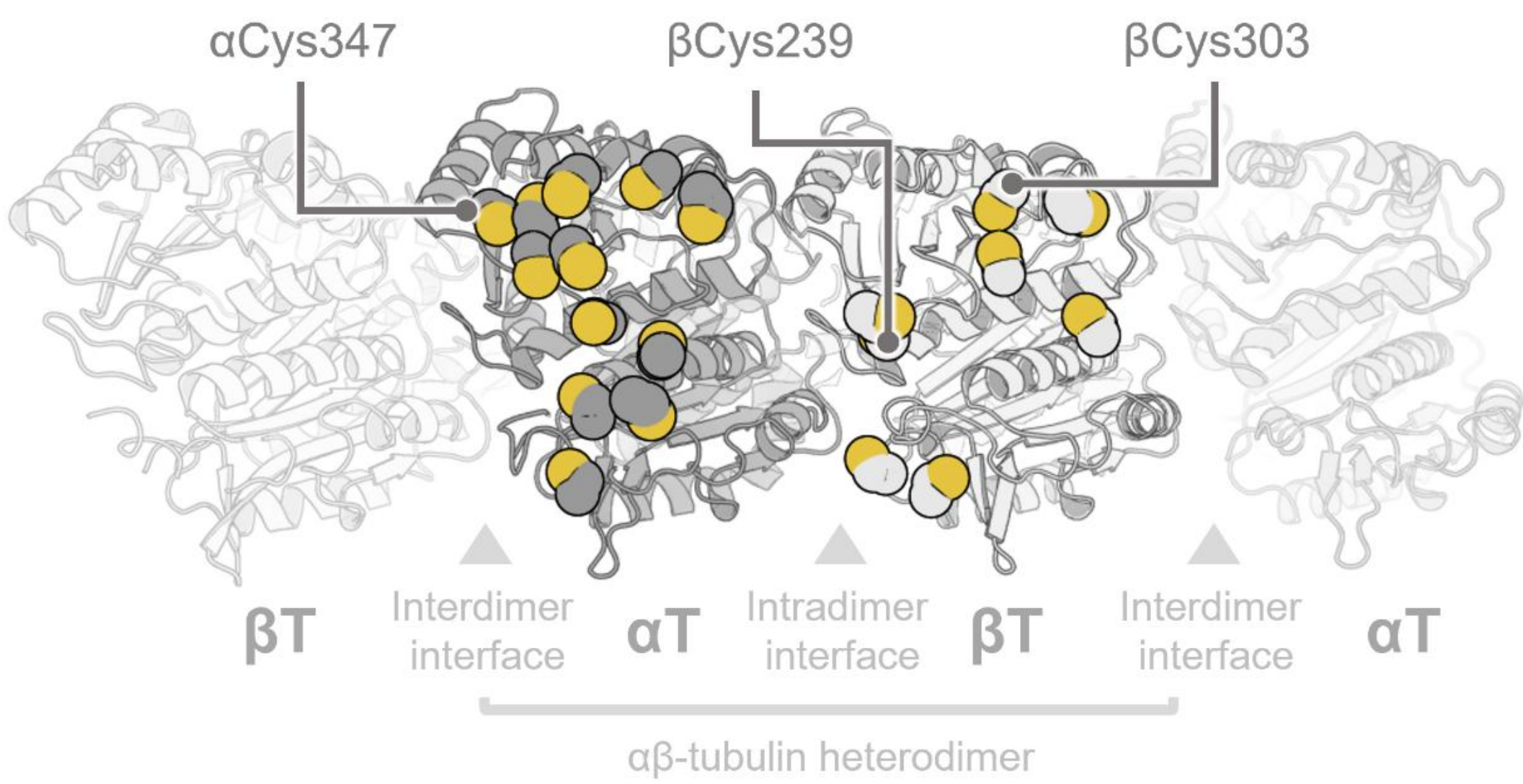

Figure S8. Depiction of a tubulin heterotetramer showing the central αβ-tubulin heterodimer in longitudinal contact with adjacent β-tubulin (βT) and α-tubulin (αT) subunits within the protofilament. Cysteine residues in the α- and β-tubulin monomers of the central heterodimer are shown as spheres, with αCys347, βCys239, and βCys303 highlighted. The intra- and interdimer interfaces are indicated. The structural model of the heterotetramer was obtained from the cryo-EM structure with PDB code 9V10[77].

III. Supplementary References

(1) Ravelli, R. B. G.; Gigant, B.; Curmi, P. A.; Jourdain, I.; Lachkar, S.; Sobel, A.; Knossow, M. Insight into Tubulin Regulation from a Complex with Colchicine and a Stathmin-like Domain. *Nature* **2004**, *428* (6979), 198–202. https://doi.org/10.1038/nature02393.

(2) Yan, W.; Yang, T.; Yang, J.; Wang, T.; Yu, Y.; Wang, Y.; Chen, Q.; Bai, P.; Li, D.; Ye, H.; Qiu, Q.; Zhou, Y.; Hu, Y.; Yang, S.; Wei, Y.; Li, W.; Chen, L. SKLB060 Reversibly Binds to Colchicine Site of Tubulin and Possesses Efficacy in Multidrug-Resistant Cell Lines. *Cell. Physiol. Biochem.* **2018**, *47* (2), 489–504. https://doi.org/10.1159/000489983.

(3) Banerjee, S.; Mahmud, F.; Deng, S.; Ma, L.; Yun, M. K.; Fakayode, S. O.; Arnst, K. E.; Yang, L.; Chen, H.; Wu, Z.; Lukka, P. B.; Parmar, K.; Meibohm, B.; White, S. W.; Wang, Y.; Li, W.; Miller, D. D. X-Ray Crystallography-Guided Design, Antitumor Efficacy, and QSAR Analysis of Metabolically Stable Cyclopenta-Pyrimidinyl Dihydroquinoxalinone as a Potent Tubulin Polymerization Inhibitor. *J. Med. Chem.* **2021**, *64* (17), 13072–13095. https://doi.org/10.1021/acs.jmedchem.1c01202.

(4) Chen, H.; Deng, S.; Albadari, N.; Yun, M. K.; Zhang, S.; Li, Y.; Ma, D.; Parke, D. N.; Yang, L.; Seagroves, T. N.; White, S. W.; Miller, D. D.; Li, W. Design, Synthesis, and Biological Evaluation of Stable Colchicine-Binding Site Tubulin Inhibitors 6-Aryl-2-Benzoyl-Pyridines as Potential Anticancer Agents. *J. Med. Chem.* **2021**, *64* (16), 12049–12074. https://doi.org/10.1021/acs.jmedchem.1c00715.

(5) Dorléans, A.; Gigant, B.; Ravelli, R. B. G.; Mailliet, P.; Mikol, V.; Knossow, M. Variations in the Colchicine-Binding Domain Provide Insight into the Structural Switch of Tubulin. *Proc. Natl. Acad. Sci. U.S.A.* **2009**, *106* (33), 13775–13779. https://doi.org/10.1073/pnas.0904223106.

(6) Niu, L.; Wang, Y.; Wang, C.; Wang, Y.; Jiang, X.; Ma, L.; Wu, C.; Yu, Y.; Chen, Q. Structure of 4′-Demethylepipodophyllotoxin in Complex with Tubulin Provides a Rationale for Drug Design. *Biochem. Biophys. Res. Commun.* **2017**, *493* (1), 718–722. https://doi.org/10.1016/j.bbrc.2017.08.125.

(7) Oliva, M. A.; Prota, A. E.; Rodríguez-Salarichs, J.; Bennani, Y. L.; Jiménez-Barbero, J.; Bargsten, K.; Canales, Á.; Steinmetz, M. O.; Díaz, J. F. Structural Basis of Noscapine Activation for Tubulin Binding. *J. Med. Chem.* **2020**, *63* (15), 8495–8501. https://doi.org/10.1021/acs.jmedchem.0c00855.

(8) Barbier, P.; Dorléans, A.; Devred, F.; Sanz, L.; Allegro, D.; Alfonso, C.; Knossow, M.; Peyrot, V.; Andreu, J. M. Stathmin and Interfacial Microtubule Inhibitors Recognize a Naturally Curved Conformation of Tubulin Dimers. *J. Biol. Chem.* **2010**, *285* (41), 31672–31681. https://doi.org/10.1074/jbc.M110.141929.

(9) Cheng, J.; Wu, Y.; Wang, Y.; Wang, C.; Wang, Y.; Wu, C.; Zeng, S.; Yu, Y.; Chen, Q. Structure of a Benzylidene Derivative of 9(10H)-Anthracenone in Complex with Tubulin Provides a Rationale for Drug Design. *Biochem. Biophys. Res. Commun.* **2018**, *495* (1), 185–188. https://doi.org/10.1016/j.bbrc.2017.10.104.

(10) Yong, C.; Devine, S. M.; Abel, A. C.; Tomlins, S. D.; Muthiah, D.; Gao, X.; Callaghan, R.; Steinmetz, M. O.; Prota, A. E.; Capuano, B.; Scammells, P. J. 1,3-Benzodioxole-Modified Noscapine Analogues: Synthesis, Antiproliferative Activity, and Tubulin-Bound Structure. *ChemMedChem* **2021**, *16* (18), 2882–2894. https://doi.org/10.1002/cmdc.202100363.

(11) Yang, J.; Yan, W.; Yu, Y.; Wang, Y.; Yang, T.; Xue, L.; Yuan, X.; Long, C.; Liu, Z.; Chen, X.; Hu, M.; Zheng, L.; Qiu, Q.; Pei, H.; Li, D.; Wang, F.; Bai, P.; Wen, J.; Ye, H.; Chen, L. The Compound Millepachine and Its Derivatives Inhibit Tubulin Polymerization by Irreversibly Binding to the Colchicine-Binding Site in β-Tubulin. *J. Biol. Chem.* **2018**, *293* (24), 9461–9472. https://doi.org/10.1074/jbc.RA117.001658.

(12) Wang, L.; Zheng, Y.; Li, D.; Yang, J.; Lei, L.; Yan, W.; Zheng, W.; Tang, M.; Shi, M.; Zhang, R.; Cai, X.; Ni, H.; Ma, X.; Li, N.; Hong, F.; Ye, H.; Chen, L. Design, Synthesis, and Bioactivity Evaluation of Dual-Target Inhibitors of Tubulin and Src Kinase Guided by Crystal Structure. *J. Med. Chem.* **2021**, *64* (12), 8127–8141. https://doi.org/10.1021/acs.jmedchem.0c01961.

(13) Prota, A. E.; Danel, F.; Bachmann, F.; Bargsten, K.; Buey, R. M.; Pohlmann, J.; Reinelt, S.; Lane, H.; Steinmetz, M. O. The Novel Microtubule-Destabilizing Drug BAL27862 Binds to the Colchicine Site of Tubulin with Distinct Effects on Microtubule Organization. *J. Mol. Biol.* **2014**, *426* (8), 1848–1860. https://doi.org/10.1016/j.jmb.2014.02.005.

(14) Fu, Z.; Hou, Y.; Ji, C.; Ma, M.; Tian, Z.; Deng, M.; Zhong, L.; Chu, Y.; Li, W. Design, Synthesis and Biological Evaluation of Anti-Pancreatic Cancer Activity of Plinabulin Derivatives Based on the Co-Crystal Structure. *Bioorg. Med. Chem.* **2018**, *26* (8), 2061–2072. https://doi.org/10.1016/j.bmc.2018.03.005.

(15) Yang, J.; Li, Y.; Qiu, Q.; Wang, R.; Yan, W.; Yu, Y.; Niu, L.; Pei, H.; Wei, H.; Ouyang, L.; Ye, H.; Xu, D.; Wei, Y.; Chen, Q.; Chen, L. Small Molecules Promote Selective Denaturation and Degradation of Tubulin Heterodimers through a Low-Barrier Hydrogen Bond. *J. Med. Chem.* **2022**, *65* (13), 9159–9173. https://doi.org/10.1021/acs.jmedchem.2c00379.

(16) McNamara, D. E.; Senese, S.; Yeates, T. O.; Torres, J. Z. Structures of Potent Anticancer Compounds Bound to Tubulin. *Protein Sci.* **2015**, *24* (7), 1164–1172. https://doi.org/10.1002/pro.2704.

(17) Ning, N.; Yu, Y.; Wu, M.; Zhang, R.; Zhang, T.; Zhu, C.; Huang, L.; Yun, C. H.; Benes, C. H.; Zhang, J.; Deng, X.; Chen, Q.; Ren, R. A Novel Microtubule Inhibitor Overcomes Multidrug Resistance in Tumors. *Cancer Res.* **2018**, *78* (20), 5949–5957. https://doi.org/10.1158/0008-5472.CAN-18-0455.

(18) Wang, Y.; Zhang, H.; Gigant, B.; Yu, Y.; Wu, Y.; Chen, X.; Lai, Q.; Yang, Z.; Chen, Q.; Yang, J. Structures of a Diverse Set of Colchicine Binding Site Inhibitors in Complex with Tubulin Provide a Rationale for Drug Discovery. *FEBS J.* **2016**, *283* (1), 102–111. https://doi.org/10.1111/febs.13555.

(19) Jiang, J.; Zhang, H.; Wang, C.; Zhang, Q.; Fang, S.; Zhou, R.; Hu, J.; Zhu, J.; Zhou, Y.; Luo, C.; Zheng, C. 1-Phenyl-Dihydrobenzoindazoles as Novel Colchicine Site Inhibitors: Structural Basis and Antitumor Efficacy. *Eur. J. Med. Chem.* **2019**, *177*, 448–456. https://doi.org/10.1016/j.ejmech.2019.04.040.

(20) Peng, Y.; Zhang, Y.; Fang, R.; Jiang, H.; Lan, G.; Xu, Z.; Liu, Y.; Nie, Z.; Ren, L.; Wang, F.; Zhang, S. D.; Ma, Y.; Yang, P.; Ge, H. H.; Zhang, W. D.; Luo, C.; Li, A.; He, W. Target Identification and Mechanistic Characterization of Indole Terpenoid Mimics: Proper Spindle Microtubule Assembly Is Essential for Cdh1-Mediated Proteolysis of CENP-A. *Adv. Sci.* **2024**, *11*, *2305593*. https://doi.org/10.1002/advs.202305593.

(21) Lai, Q.; Wang, Y.; Wang, R.; Lai, W.; Tang, L.; Tao, Y.; Liu, Y.; Zhang, R.; Huang, L.; Xiang, H.; Zeng, S.; Gou, L.; Chen, H.; Yao, Y.; Yang, J. Design, Synthesis and Biological Evaluation of a Novel Tubulin Inhibitor 7a3 Targeting the Colchicine Binding Site. *Eur. J. Med. Chem.* **2018**, *156*, 162–179. https://doi.org/10.1016/j.ejmech.2018.05.010.

(22) Li, Z.; Ma, L.; Wu, C.; Meng, T.; Ma, L.; Zheng, W.; Yu, Y.; Chen, Q.; Yang, J.; Shen, J. The Structure of MT189-Tubulin Complex Provides Insights into Drug Design. *Lett. Drug Des. Discov.* **2019**, *16* (9), 1069–1073. https://doi.org/10.2174/1570180816666181122122655.

(23) Xian, J.; Bu, F.; Wang, Y.; Long, F.; Zhang, Z.; Wu, C.; Tao, Y.; Wang, T.; Wang, G. A Rationale for Drug Design Provided by Co-Crystal Structure of IC261 in Complex with Tubulin. *Molecules* **2021**, *26* (4), 946. https://doi.org/10.3390/molecules26040946.

(24) Chen, H.; Deng, S.; Wang, Y.; Albadari, N.; Kumar, G.; Ma, D.; Li, W.; White, S. W.; Miller, D. D.; Li, W. Structure-Activity Relationship Study of Novel 6-Aryl-2-Benzoyl-Pyridines as Tubulin Polymerization Inhibitors with Potent Antiproliferative Properties. *J. Med. Chem.* **2020**, *63* (2), 827–846. https://doi.org/10.1021/acs.jmedchem.9b01815.

(25) Ren, Y.; Wang, Y.; Li, G.; Zhang, Z.; Ma, L.; Cheng, B.; Chen, J. Discovery of Novel Benzimidazole and Indazole Analogues as Tubulin Polymerization Inhibitors with Potent Anticancer Activities. *J. Med. Chem.* **2021**, *64* (8), 4498–4515. https://doi.org/10.1021/acs.jmedchem.0c01837.

(26) Zhou, P.; Liu, Y.; Zhou, L.; Zhu, K.; Feng, K.; Zhang, H.; Liang, Y.; Jiang, H.; Luo, C.; Liu, M.; Wang, Y. Potent Antitumor Activities and Structure Basis of the Chiral β-Lactam Bridged Analogue of Combretastatin A-4 Binding to Tubulin. *J. Med. Chem.* **2016**, *59* (22), 10329–10334. https://doi.org/10.1021/acs.jmedchem.6b01268.

(27) Banerjee, S.; Arnst, K. E.; Wang, Y.; Kumar, G.; Deng, S.; Yang, L.; Li, G. B.; Yang, J.; White, S. W.; Li, W.; Miller, D. D. Heterocyclic-Fused Pyrimidines as Novel Tubulin Polymerization Inhibitors Targeting the Colchicine Binding Site: Structural Basis and Antitumor Efficacy. *J. Med. Chem.* **2018**, *61* (4), 1704–1718. https://doi.org/10.1021/acs.jmedchem.7b01858.

(28) Ma, L.; Lei, J.; Chen, H.; Huang, R.; Su, T.; Feng, J.; Sun, Q. Structural Insight into SSE15206 in Complex With Tubulin Provides a Rational Design for Pyrazolinethioamides as Tubulin Polymerization Inhibitors. *Future Med. Chem.* **2022**, *14* (11), 785–794. https://doi.org/10.4155/fmc-2021-0124.

(29) Arnst, K. E.; Wang, Y.; Hwang, D. J.; Xue, Y.; Costello, T.; Hamilton, D.; Chen, Q.; Yang, J.; Park, F.; Dalton, J. T.; Miller, D. D.; Li, W. A Potent, Metabolically Stable Tubulin Inhibitor Targets the Colchicine Binding Site and Overcomes Taxane Resistance. *Cancer Res.* **2018**, *78* (1), 265–277. https://doi.org/10.1158/0008-5472.CAN-17-0577.

(30) Lei, J. H.; Ma, L. L.; Xian, J. H.; Chen, H.; Zhou, J. J.; Chen, H.; Lei, Q.; Li, Y. Y.; Wang, Y. Y.; Wang, Y. X. Structural Insights into Targeting of the Colchicine Binding Site by ELR510444 and Parbendazole to Achieve Rational Drug Design. *RSC Adv.* **2021**, *11* (31), 18938–18944. https://doi.org/10.1039/d1ra01173a.

(31) Zhao, W.; Zhou, C.; Guan, Z.-Y.; Yin, P.; Chen, F.; Tang, Y. J. Structural Insights into the Inhibition of Tubulin by the Antitumor Agent 4β-(1,2,4-Triazol-3-Ylthio)-4-Deoxypodophyllotoxin. *ACS Chem. Biol.* **2017**, *12* (3), 746–752. https://doi.org/10.1021/acschembio.6b00842.

(32) Canela, M.-D.; Noppen, S.; Bueno, O.; Prota, A. E.; Bargsten, K.; Sáez-Calvo, G.; Jimeno, M.; Benkheil, M.; Ribatti, D.; Velázquez, S.; Camarasa, M.-J.; Díaz, J. F.; Steinmetz, M. O.; Priego, E.; Pérez-Pérez, M.-J.; Liekens, S. Antivascular and Antitumor Properties of the Tubulin-Binding Chalcone TUB091. *Oncotarget* **2017**, *8* (9), 14325–14342. https://doi.org/10.18632/oncotarget.9527.

(33) Yan, W.; Li, Y.; Liu, Y.; Wen, Y.; Pei, H.; Yang, J.; Chen, L. Crystal Structure of Tubulin-Barbigerone Complex Enables Rational Design of Potent Anticancer Agents with Isoflavone Skeleton. *Phytomedicine* **2023**, *109*, 154550. https://doi.org/10.1016/j.phymed.2022.154550.

(34) Marangon, J.; Christodoulou, M. S.; Casagrande, F. V. M.; Tiana, G.; Dalla Via, L.; Aliverti, A.; Passarella, D.; Cappelletti, G.; Ricagno, S. Tools for the Rational Design of Bivalent Microtubule-Targeting Drugs. *Biochem. Biophys. Res. Commun.* **2016**, *479* (1), 48–53. https://doi.org/10.1016/j.bbrc.2016.09.022.

(35) Wang, Q.; Arnst, K. E.; Wang, Y.; Kumar, G.; Ma, D.; Chen, H.; Wu, Z.; Yang, J.; White, S. W.; Miller, D. D.; Li, W. Structural Modification of the 3,4,5-Trimethoxyphenyl Moiety in the Tubulin Inhibitor VERU-111 Leads to Improved Antiproliferative Activities. *J. Med. Chem.* **2018**, *61* (17), 7877–7891. https://doi.org/10.1021/acs.jmedchem.8b00827.

(36) Li, Y.; Liu, Y.; Zhu, Z.; Yan, W.; Zhang, C.; Yang, Z.; Bai, P.; Tang, M.; Shi, M.; He, W.; Fu, S.; Liu, J.; Han, K.; Li, J.; Xie, L.; Ye, H.; Yang, J.; Chen, L. Structure-Based Design and Synthesis of N-Substituted 3-Amino-β-Carboline Derivatives as Potent αβ-Tubulin Degradation Agents. *J. Med. Chem.* **2022**, *65* (3), 2675–2693. https://doi.org/10.1021/acs.jmedchem.1c02159.

(37) Gaspari, R.; Prota, A. E.; Bargsten, K.; Cavalli, A.; Steinmetz, M. O. Structural Basis of Cis- and Trans-Combretastatin Binding to Tubulin. *Chem* **2017**, *2* (1), 102–113. https://doi.org/10.1016/j.chempr.2016.12.005.

(38) Arnst, K. E.; Banerjee, S.; Wang, Y.; Chen, H.; Li, Y.; Yang, L.; Li, W.; Miller, D. D.; Li, W. X-Ray Crystal Structure-Guided Discovery and Antitumor Efficacy of Dihydroquinoxalinone as Potent Tubulin Polymerization Inhibitors. *ACS Chem. Biol.* **2019**, *14* (12), 2810–2821. https://doi.org/10.1021/acschembio.9b00696.

(39) Bohnacker, T.; Prota, A. E.; Beaufils, F.; Burke, J. E.; Melone, A.; Inglis, A. J.; Rageot, D.; Sele, A. M.; Cmiljanovic, V.; Cmiljanovic, N.; Bargsten, K.; Aher, A.; Akhmanova, A.; Díaz, J. F.; Fabbro, D.; Zvelebil, M.; Williams, R. L.; Steinmetz, M. O.; Wymann, M. P. Deconvolution of Buparlisib's Mechanism of Action Defines Specific PI3K and Tubulin Inhibitors for Therapeutic Intervention. *Nat. Commun.* **2017**, *8*, 14683. https://doi.org/10.1038/ncomms14683.

(40) Cury, N. M.; Mühlethaler, T.; Laranjeira, A. B. A.; Canevarolo, R. R.; Zenatti, P. P.; Lucena-Agell, D.; Barasoain, I.; Song, C.; Sun, D.; Dovat, S.; Yunes, R. A.; Prota, A. E.; Steinmetz, M. O.; Díaz, J. F.; Yunes, J. A. Structural Basis of Colchicine-Site Targeting Acylhydrazones Active against Multidrug-Resistant Acute Lymphoblastic Leukemia. *iScience* **2019**, *21*, 95–109. https://doi.org/10.1016/j.isci.2019.10.003.

(41) Bueno, O.; Estévez Gallego, J.; Martins, S.; Prota, A. E.; Gago, F.; Gómez-Sanjuan, A.; Camarasa, M. J.; Barasoain, I.; Steinmetz, M. O.; Díaz, J. F.; Pérez-Pérez, M. J.; Liekens, S.; Priego, E. M. High-Affinity Ligands of the Colchicine Domain in Tubulin Based on a Structure-Guided Design. *Sci. Rep.* **2018**, *8*, 22382. https://doi.org/10.1038/s41598-018-22382-x.

(42) Oliva, M. Á.; Tosat-Bitrián, C.; Barrado-Gil, L.; Bonato, F.; Galindo, I.; Garaigorta, U.; Álvarez-Bernad, B.; París-Ogáyar, R.; Lucena-Agell, D.; Giménez-Abián, J. F.; García-Dorival, I.; Urquiza, J.; Gastaminza, P.; Díaz, J. F.; Palomo, V.; Alonso, C. Effect of Clinically Used Microtubule Targeting Drugs on Viral Infection and Transport Function. *Int. J. Mol. Sci.* **2022**, *23* (7), 3448. https://doi.org/10.3390/ijms23073448.

(43) Pochampally, S.; Hartman, K. L.; Wang, R.; Wang, J.; Yun, M. K.; Parmar, K.; Park, H.; Meibohm, B.; White, S. W.; Li, W.; Miller, D. D. Design, Synthesis, and Biological Evaluation of Pyrimidine Dihydroquinoxalinone Derivatives as Tubulin Colchicine Site-Binding Agents That Displayed Potent Anticancer Activity Both In Vitro and In Vivo. *ACS Pharmacol. Transl. Sci.* **2023**, *6* (4), 526–545. https://doi.org/10.1021/acsptsci.2c00108.

(44) Smedley, C. J.; Stanley, P. A.; Qazzaz, M. E.; Prota, A. E.; Olieric, N.; Collins, H.; Eastman, H.; Barrow, A. S.; Lim, K.-H.; Kam, T.-S.; Smith, B. J.; Duivenvoorden, H. M.; Parker, B. S.; Bradshaw, T. D.; Steinmetz, M. O.; Moses, J. E. Sustainable Syntheses of (−)-Jerantinines A & E and Structural Characterisation of the Jerantinine-Tubulin Complex at the Colchicine Binding Site. *Sci. Rep.* **2018**, *8* (1), 10617. https://doi.org/10.1038/s41598-018-28880-2.

(45) Field, J. J.; Pera, B.; Gallego, J. E.; Calvo, E.; Rodríguez-Salarichs, J.; Sáez-Calvo, G.; Zuwerra, D.; Jordi, M.; Andreu, J. M.; Prota, A. E.; Ménchon, G.; Miller, J. H.; Altmann, K. H.; Díaz, J. F. Zampanolide Binding to Tubulin Indicates Cross-Talk of Taxane Site with Colchicine and Nucleotide Sites. *J. Nat. Prod.* **2018**, *81* (3), 494–505. https://doi.org/10.1021/acs.jnatprod.7b00704.

(46) Brindisi, M.; Ulivieri, C.; Alfano, G.; Gemma, S.; de Asís Balaguer, F.; Khan, T.; Grillo, A.; Chemi, G.; Menchon, G.; Prota, A. E.; Olieric, N.; Lucena-Agell, D.; Barasoain, I.; Diaz, J. F.; Nebbioso, A.; Conte, M.; Lopresti, L.; Magnano, S.; Amet, R.; Kinsella, P.; Zisterer, D. M.; Ibrahim, O.; O'Sullivan, J.; Morbidelli, L.; Spaccapelo, R.; Baldari, C.; Butini, S.; Novellino, E.; Campiani, G.; Altucci, L.; Steinmetz, M. O.; Brogi, S. Structure-Activity Relationships, Biological Evaluation and Structural Studies of Novel Pyrrolonaphthoxazepines as Antitumor Agents. *Eur. J. Med. Chem.* **2019**, *162*, 290–320. https://doi.org/10.1016/j.ejmech.2018.11.004.

(47) Sharma, A.; Gonzalo, S.; Olieric, N.; As, F. De. Quinolin-6-Yloxyacetamides Are Microtubule Destabilizing Agents That Bind to the Colchicine Site of Tubulin. *Int. J. Mol. Sci.* **2017**, *18*, 1336–1347. https://doi.org/10.3390/ijms18071336.

(48) Naret, T.; Khelifi, I.; Provot, O.; Bignon, J.; Levaique, H.; Dubois, J.; Souce, M.; Kasselouri, A.; Deroussent, A.; Paci, A.; Varela, P. F.; Gigant, B.; Alami, M.; Hamze, A. 1,1-Diheterocyclic Ethylenes Derived from Quinaldine and Carbazole as New Tubulin-Polymerization Inhibitors: Synthesis, Metabolism, and Biological Evaluation. *J. Med. Chem.* **2019**, *62* (4), 1902–1916. https://doi.org/10.1021/acs.jmedchem.8b01386.

(49) Zhang, J.; Tan, L.; Wu, C.; Li, Y.; Chen, H.; Liu, Y.; Wang, Y. Discovery and Biological Evaluation of 4,6-Pyrimidine Analogues with Potential Anticancer Agents as Novel Colchicine Binding Site Inhibitors. *Eur. J. Med. Chem.* **2023**, *248*, 115085. https://doi.org/10.1016/j.ejmech.2022.115085.

(50) Dohle, W.; Jourdan, F. L.; Menchon, G.; Prota, A. E.; Foster, P. A.; Mannion, P.; Hamel, E.; Thomas, M. P.; Kasprzyk, P. G.; Ferrandis, E.; Steinmetz, M. O.; Leese, M. P.; Potter, B. V. L. Quinazolinone-Based Anticancer Agents: Synthesis, Antiproliferative SAR, Antitubulin Activity, and Tubulin Co-Crystal Structure. *J. Med. Chem.* **2018**, *61* (3), 1031–1044. https://doi.org/10.1021/acs.jmedchem.7b01474.

(51) Dohle, W.; Prota, A. E.; Menchon, G.; Hamel, E.; Steinmetz, M. O.; Potter, B. V. L. Tetrahydroisoquinoline Sulfamates as Potent Microtubule Disruptors: Synthesis, Antiproliferative and Antitubulin Activity of Dichlorobenzyl-Based Derivatives, and a Tubulin Co-crystal Structure. *ACS Omega* **2019**, *4* (1), 755–764. https://doi.org/10.1021/acsomega.8b02879.

(52) Jost, M.; Chen, Y.; Gilbert, L. A.; Horlbeck, M. A.; Krenning, L.; Menchon, G.; Rai, A.; Cho, M. Y.; Stern, J. J.; Prota, A. E.; Kampmann, M.; Akhmanova, A.; Steinmetz, M. O.; Tanenbaum, M. E.; Weissman, J. S. Combined CRISPRi/a-Based Chemical Genetic Screens Reveal That Rigosertib Is a Microtubule-Destabilizing Agent. *Mol. Cell* **2017**, *68* (1), 210–223.e6. https://doi.org/10.1016/j.molcel.2017.09.012.

(53) Zhang, Z.; Wang, C.; Ma, L.; Jiang, X.; Wu, C.; Wang, Y.; Jiang, Y.; Zheng, W.; Yang, Y.; Ma, Y.; Yang, J. Molecular Mechanism of Crolibulin in Complex with Tubulin Provides a Rationale for Drug Design. *Biochem. Biophys. Res. Commun.* **2019**, *511* (2), 381–386. https://doi.org/10.1016/j.bbrc.2019.02.064.

(54) Mühlethaler, T.; Gioia, D.; Prota, A. E.; Sharpe, M. E.; Cavalli, A.; Steinmetz, M. O. Comprehensive Analysis of Binding Sites in Tubulin. *Angew. Chem. Int. Ed.* **2021**, *60* (24), 13331–13342. https://doi.org/10.1002/anie.202100273.

(55) Li, Y.; Yang, J.; Niu, L.; Hu, D.; Li, H.; Chen, L.; Yu, Y.; Chen, Q. Structural Insights into the Design of Indole Derivatives as Tubulin Polymerization Inhibitors. *FEBS Lett.* **2020**, *594* (1), 199–204. https://doi.org/10.1002/1873-3468.13566.

(56) Tan, L.; Wu, C.; Zhang, J.; Yu, Q.; Wang, X.; Zhang, L.; Ge, M.; Wang, Z.; Ouyang, L.; Wang, Y. Design, Synthesis, and Biological Evaluation of Heterocyclic-Fused Pyrimidine Chemotypes Guided by X-Ray Crystal Structure with Potential Antitumor and Anti-Multidrug Resistance Efficacy Targeting the Colchicine Binding Site. *J. Med. Chem.* **2023**, *66* (5), 3588–3620. https://doi.org/10.1021/acs.jmedchem.2c02115.

(57) Niu, L.; Yang, J.; Yan, W.; Yu, Y.; Zheng, Y.; Ye, H.; Chen, Q.; Chen, L. Reversible Binding of the Anticancer Drug KXO1 (Tirbanibulin) to the Colchicine-Binding Site of β-Tubulin Explains KXO1's Low Clinical Toxicity. *J. Biol. Chem.* **2019**, *294* (48), 18099–18108. https://doi.org/10.1074/jbc.RA119.010732.

(58) Wang, T.; Wu, C.; Wang, C.; Zhang, G.; Arnst, K. E.; Yao, Y.; Zhang, Z.; Wang, Y.; Pu, D.; Li, W. Unraveling the Molecular Mechanism of BNC105, a Phase II Clinical Trial Vascular Disrupting Agent, Provides Insights into Drug Design. *Biochem. Biophys. Res. Commun.* **2020**, *525* (1), 148–154. https://doi.org/10.1016/j.bbrc.2019.12.083.

(59) Du, T.; Lin, S.; Ji, M.; Xue, N.; Liu, Y.; Zhang, Z. A Novel Orally Active Microtubule Destabilizing Agent S-40 Targets the Colchicine-Binding Site and Shows Potent Antitumor Activity. *Cancer Lett.* **2020**, *495*, 22–32. https://doi.org/10.1016/j.canlet.2020.08.040.

(60) Li, G.; Wang, Y.; Li, L.; Ren, Y.; Deng, X.; Liu, J.; Wang, W.; Luo, M.; Liu, S.; Chen, J. Design, Synthesis, and Bioevaluation of Pyrazolo[1,5-a]Pyrimidine Derivatives as Tubulin Polymerization Inhibitors Targeting the Colchicine Binding Site with Potent Anticancer Activities. *Eur. J. Med. Chem.* **2020**, *202*, 112519. https://doi.org/10.1016/j.ejmech.2020.112519.

(61) Lin, S.; Du, T.; Zhang, J.; Wu, D.; Tian, H.; Zhang, K.; Jiang, L.; Lu, D.; Sheng, L.; Li, Y.; Ji, M.; Chen, X.; Xu, H. Optimization of Benzamide Derivatives as Potent and Orally Active Tubulin Inhibitors Targeting the Colchicine Binding Site. *J. Med. Chem.* **2022**, *65* (24), 16372–16391. https://doi.org/10.1021/acs.jmedchem.2c01208.

(62) Wranik, M.; Weinert, T.; Slavov, C.; Masini, T.; Furrer, A.; Gaillard, N.; Gioia, D.; Ferrarotti, M.; James, D.; Glover, H.; Carrillo, M.; Kekilli, D.; Stipp, R.; Skopintsev, P.; Brünle, S.; Mühlethaler, T.; Beale, J.; Gashi, D.; Nass, K.; Ozerov, D.; Johnson, P. J. M.; Cirelli, C.; Bacellar, C.; Braun, M.; Wang, M.; Dworkowski, F.; Milne, C.; Cavalli, A.; Wachtveitl, J.; Steinmetz, M. O.; Standfuss, J. Watching the Release of a Photopharmacological Drug from Tubulin Using Time-Resolved Serial Crystallography. *Nat. Commun.* **2023**, *14*, 903. https://doi.org/10.1038/s41467-023-36481-5.

(63) Gao, L.; Meiring, J. C. M.; Varady, A.; Ruider, I. E.; Heise, C.; Wranik, M.; Velasco, C. D.; Taylor, J. A.; Terni, B.; Weinert, T.; Standfuss, J.; Cabernard, C. C.; Llobet, A.; Steinmetz, M. O.; Bausch, A. R.; Distel, M.; Thorn-Seshold, J.; Akhmanova, A.; Thorn-Seshold, O. In Vivo Photocontrol of Microtubule Dynamics and Integrity, Migration and Mitosis, by the Potent GFP-Imaging-Compatible Photoswitchable Reagents SBTubA4P and SBTub2M. *J. Am. Chem. Soc.* **2022**, *144* (12), 5614–5628. https://doi.org/10.1021/jacs.2c01020.

(64) Arnst, K. E.; Wang, Y.; Lei, Z. N.; Hwang, D. J.; Kumar, G.; Ma, D.; Parke, D. N.; Chen, Q.; Yang, J.; White, S. W.; Seagroves, T. N.; Chen, Z. S.; Miller, D. D.; Li, W. Colchicine Binding Site Agent DJ95 Overcomes Drug Resistance and Exhibits Antitumor Efficacy. *Mol. Pharmacol.* **2019**, *96* (1), 73–89. https://doi.org/10.1124/mol.118.114801.

(65) Wang, Q.; Arnst, K. E.; Wang, Y.; Kumar, G.; Ma, D.; White, S. W.; Miller, D. D.; Li, W.; Li, W. Structure-Guided Design, Synthesis, and Biological Evaluation of (2-(1 H-Indol-3-Yl)-1 H-Imidazol-4-Yl)(3,4,5-Trimethoxyphenyl) Methanone (ABI-231) Analogues Targeting the Colchicine Binding Site in Tubulin. *J. Med. Chem.* **2019**, *62* (14), 6734–6750. https://doi.org/10.1021/acs.jmedchem.9b00706.

(66) de la Roche, N. M.; Mühlethaler, T.; Di Martino, R. M. C.; Ortega, J. A.; Gioia, D.; Roy, B.; Prota, A. E.; Steinmetz, M. O.; Cavalli, A. Novel Fragment-Derived Colchicine-Site Binders as Microtubule-Destabilizing Agents. *Eur. J. Med. Chem.* **2022**, *241*, 114614. https://doi.org/10.1016/j.ejmech.2022.114614.

(67) Lucena-Agell, D.; Guillén, M. J.; Matesanz, R.; Álvarez-Bernad, B.; Hortigüela, R.; Avilés, P.; Martínez-Díez, M.; Santamaría-Núñez, G.; Contreras, J.; Plaza-Menacho, I.; Giménez-Abián, J. F.; Oliva, M. A.; Cuevas, C.; Díaz, J. F. PM534,

an Optimized Target-Protein Interaction Strategy through the Colchicine Site of Tubulin. *J. Med. Chem.* **2024**, *67* (4), 2619–2630. https://doi.org/10.1021/acs.jmedchem.3c01775.

(68) Barreca, M.; Spanò, V.; Rocca, R.; Bivacqua, R.; Abel, A. C.; Maruca, A.; Montalbano, A.; Raimondi, M. V.; Tarantelli, C.; Gaudio, E.; Cascione, L.; Rinaldi, A.; Bai, R.; Steinmetz, M. O.; Prota, A. E.; Alcaro, S.; Hamel, E.; Bertoni, F.; Barraja, P. Development of [1,2]Oxazoloisoindoles Tubulin Polymerization Inhibitors: Further Chemical Modifications and Potential Therapeutic Effects against Lymphomas. *Eur. J. Med. Chem.* **2022**, *243*, 114744. https://doi.org/10.1016/j.ejmech.2022.114744.

(69) Patterson, J. C.; Joughin, B. A.; Prota, A. E.; Mühlethaler, T.; Jonas, O. H.; Whitman, M. A.; Varmeh, S.; Chen, S.; Balk, S. P.; Steinmetz, M. O.; Lauffenburger, D. A.; Yaffe, M. B. VISAGE Reveals a Targetable Mitotic Spindle Vulnerability in Cancer Cells. *Cell Syst.* **2019**, *9* (1), 74–92.e8. https://doi.org/10.1016/j.cels.2019.05.009.

(70) Zhou, P.; Liang, Y.; Zhang, H.; Jiang, H.; Feng, K.; Xu, P.; Wang, J.; Wang, X.; Ding, K.; Luo, C.; Liu, M.; Wang, Y. Design, Synthesis, Biological Evaluation and Cocrystal Structures with Tubulin of Chiral β-Lactam Bridged Combretastatin A-4 Analogues as Potent Antitumor Agents. *Eur. J. Med. Chem.* **2018**, *144*, 817–842. https://doi.org/10.1016/j.ejmech.2017.12.004.

(71) Shchegravina, E. S.; Svirshchevskaya, E. V.; Combes, S.; Allegro, D.; Barbier, P.; Gigant, B.; Varela, P. F.; Gavryushin, A. E.; Kobanova, D. A.; Shchekotikhin, A. E.; Fedorov, A. Y. Discovery of Dihydrofuranoallocolchicinoids—Highly Potent Antimitotic Agents with Low Acute Toxicity. *Eur. J. Med. Chem.* **2020**, *207*, 112724. https://doi.org/10.1016/j.ejmech.2020.112724.

(72) Jernigan, F.; Branstrom, A.; Baird, J. D.; Cao, L.; Dali, M.; Furia, B.; Kim, M. J.; O'Keefe, K.; Kong, R.; Laskin, O. L.; Colacino, J. M.; Pykett, M.; Mollin, A.; Sheedy, J.; Dumble, M.; Moon, Y. C.; Sheridan, R.; Mühlethaler, T.; Spiegel, R. J.; Prota, A. E.; Steinmetz, M. O.; Weetall, M. Preclinical and Early Clinical Development of PTC596, a Novel Small-Molecule Tubulin-Binding Agent. *Mol. Cancer Ther.* **2021**, *20* (10), 1846–1857. https://doi.org/10.1158/1535-7163.MCT-20-0774.

(73) Stein, A.; Hilken née Thomopoulou, P.; Frias, C.; Hopff, S. M.; Varela, P.; Wilke, N.; Mariappan, A.; Neudörfl, J. M.; Fedorov, A. Y.; Gopalakrishnan, J.; Gigant, B.; Prokop, A.; Schmalz, H. G. B-nor-Methylene Colchicinoid PT-100 Selectively Induces Apoptosis in Multidrug-Resistant Human Cancer Cells via an Intrinsic Pathway in a Caspase-Independent Manner. *ACS Omega* **2022**, *7* (3), 2591–2603. https://doi.org/10.1021/acsomega.1c04659.

(74) Wranik, M.; Kepa, M. W.; Beale, E. V.; James, D.; Bertrand, Q.; Weinert, T.; Furrer, A.; Glover, H.; Gashi, D.; Carrillo, M.; Kondo, Y.; Stipp, R. T.; Khusainov, G.; Nass, K.; Ozerov, D.; Cirelli, C.; Johnson, P. J. M.; Dworkowski, F.; Beale, J. H.; Stubbs, S.; Zamofing, T.; Schneider, M.; Krauskopf, K.; Gao, L.; Thorn-Seshold, O.; Bostedt, C.; Bacellar, C.; Steinmetz, M. O.; Milne, C.; Standfuss, J. A Multi-Reservoir Extruder for Time-Resolved Serial Protein Crystallography and Compound Screening at X-Ray Free-Electron Lasers. *Nat. Commun.* **2023**, *14*, 7956. https://doi.org/10.1038/s41467-023-43523-5.

(75) Ding, Z.; Ma, M.; Zhong, C.; Wang, S.; Fu, Z.; Hou, Y.; Liu, Y.; Zhong, L.; Chu, Y.; Li, F.; Song, C.; Wang, Y.; Yang, J.; Li, W. Development of Novel Phenoxy-Diketopiperazine-Type Plinabulin Derivatives as Potent Antimicrotubule Agents Based on the Co-Crystal Structure. *Bioorg. Med. Chem.* **2020**, *28* (1), 115186. https://doi.org/10.1016/j.bmc.2019.115186.

(76) Deng, S.; Banerjee, S.; Chen, H.; Pochampally, S.; Wang, Y.; Yun, M. K.; White, S. W.; Parmar, K.; Meibohm, B.; Hartman, K. L.; Wu, Z.; Miller, D. D.; Li, W. SB226, an Inhibitor of Tubulin Polymerization, Inhibits Paclitaxel-Resistant Melanoma Growth and Spontaneous Metastasis. *Cancer Lett.* **2023**, *555*, 216046. https://doi.org/10.1016/j.canlet.2022.216046.

(77) Liu, Q.; Zhou, L.; Liang, X.; Chen, P.; Li, B.; Yang, S.; Liu, Y.; Zhao, H.; Hu, J.; Feng, S.; Xie, S.; Wu, J.; Gui, M. Doublet Microtubule-Associated Tektins and Enzymes Differentially Regulate Sperm Flagellar Integrity and Motility. *Nat. Commun.* **2026**, *17*, 3316. https://doi.org/10.1038/s41467-026-69714-4.
